# Identification and functional characterization of BAHD acyltransferases associated with anthocyanin acylation in blueberry

**DOI:** 10.1093/hr/uhaf041

**Published:** 2025-02-10

**Authors:** Molla F Mengist, Muhammad Ali Abid, Mary H Grace, Romit Seth, Nahla Bassil, Colin D Kay, Andrew P Dare, David Chagné, Richard V Espley, Andrew Neilson, Mary Ann Lila, Mario Ferruzzi, Massimo Iorizzo

**Affiliations:** Plants for Human Health Institute, North Carolina State University, Kannapolis, NC 28081, USA; Agricultural Research Station, Virginia State University, Petersburg, VA 23806, USA; Plants for Human Health Institute, North Carolina State University, Kannapolis, NC 28081, USA; Plants for Human Health Institute, North Carolina State University, Kannapolis, NC 28081, USA; Plants for Human Health Institute, North Carolina State University, Kannapolis, NC 28081, USA; National Clonal Germplasm Repository, USDA-ARS, Corvallis, OR 97333, USA; Plants for Human Health Institute, North Carolina State University, Kannapolis, NC 28081, USA; Arkansas Children’s Nutrition Center (ACNC), University of Arkansas for Medical Sciences, Little Rock, AR 72202, USA; The New Zealand Institute for Plant and Food Research Limited, Auckland 92169, New Zealand; The New Zealand Institute for Plant and Food Research Limited, Palmerston North 4442, New Zealand; The New Zealand Institute for Plant and Food Research Limited, Auckland 92169, New Zealand; Plants for Human Health Institute, North Carolina State University, Kannapolis, NC 28081, USA; Department of Food, Bioprocessing and Nutrition Sciences, North Carolina State University, Raleigh, NC 27606, USA; Plants for Human Health Institute, North Carolina State University, Kannapolis, NC 28081, USA; Department of Food, Bioprocessing and Nutrition Sciences, North Carolina State University, Raleigh, NC 27606, USA; Plants for Human Health Institute, North Carolina State University, Kannapolis, NC 28081, USA; Arkansas Children’s Nutrition Center (ACNC), University of Arkansas for Medical Sciences, Little Rock, AR 72202, USA; Plants for Human Health Institute, North Carolina State University, Kannapolis, NC 28081, USA; Department of Horticultural Science, North Carolina State University, Raleigh, NC 27607, USA

## Abstract

Blueberry is promoted as a super food with several health properties derived from chlorogenic acid and anthocyanin. Previous studies indicated that anthocyanin acylation and the content of chlorogenic acid could affect their level of absorption and biological activity. In this study, a genome-wide association study was performed to identify loci associated with anthocyanin and chlorogenic acid and characterize the candidate genes controlling anthocyanin acylation. Two stable loci controlling anthocyanin acylation and glucose specific glycosylation were confirmed on chromosomes 2 and 4, respectively, while no stable loci associated with chlorogenic acid were identified. Two acyl-CoA acyltransferases named *VcBAHD-AT1* and *VcBAHD-AT4* were identified as best candidate genes controlling anthocyanin acylation. Interestingly, the two genes clustered in acyl-CoA acyltransferases clade III, a clade that is not commonly associated with anthocyanin acylation. A virus-induced gene silencing approach optimized for silencing *VcBAHD-AT1* and *VcBAHD-AT4* in the whole blueberry fruits, confirmed the role of these two genes in anthocyanin acylation. Overall, this study establishes the foundation to develop a molecular marker to select for higher acylated anthocyanin and delivered a method for rapid functional characterization of genes associated with other fruit related traits in blueberry. Also, the study adds evidence that during the evolution of acyl-CoA acyltransferases multiple routes led to the emergence and/or fixation of the anthocyanin acyltransferase activity. These outcomes advance knowledge about the genes controlling anthocyanin acylation in blueberries and that extend to other plants. Selecting new blueberry cultivars with higher acylated anthocyanin levels could potentially increase absorption of this health-related bioactive.

## Introduction

Blueberries are widely known for their health benefits, primarily associated with bioactive phytochemicals such as anthocyanin (ACN) and chlorogenic acid (CGA) [1] . Over the last decade the multitude of health effects associated with blueberry consumption have fueled a surge in blueberry consumption and production in the USA and globally [2]. Given the important role of these bioactives in blueberry, in the last ten years significant efforts have been made to understand the content, diversity, functional properties, and genetics of these phytochemicals.

Highbush blueberry varieties exhibit a complex ACN profile comprising 18–20 chemical structures, including delphinidin, cyanidin, petunidin, peonidin, and malvidin, glycosylated with different sugar moieties including arabinose (arab), galactose (galc), and glucose (gluc) [3, 4]. The addition of acyl groups to the glycosylated ACN is another typical chemical modification in some blueberry species and involves the esterification of hydroxyl groups by aliphatic acyl donors or aromatic acyl donors. The acylation of ACN contributes to the structural diversity of these pigments, influencing their stability, bioaccessibility or bioavailability, and potential health benefits [5]. For instance, a study [6] found that acylated ACN had significantly higher relative bioaccessibility than non-acylated ACN, which could lead to increased absorption (bioavailability) of these bioactive compounds. This is particularly important as the low bioavailability of blueberry ACN limits their biological activities [5]. Also, recent findings emphasized the higher color stability of acylated ACN, indicating their potential relevance as natural colorants in the food industry [7–9]. CGA has multiple health-protective properties, including recent studies showing that CGA- and CGA-rich blueberries inhibit production of gut microbial metabolites that promote atherosclerosis [10].

Phenotypic variations for ACN and CGA concentration exist within blueberry germplasm, with ACN acylation and glycosylation being the main factors to distinguish this blueberry germplasm [3, 4, 6, 11]. Recent studies by [12, 13] have unveiled the genetic basis of ACN and CGA. These investigations employed biparental linkage mapping, shedding light on the genetic basis of these traits. Notably, major QTLs associated with ACN glycosylation and acylation and potential candidate genes associated with these QTLs were identified [12, 13]. However, biparental mapping, while informative, comes with limitations, including the low mapping resolution. To address this limitation researchers integrate biparental mapping data with a genome-wide association study (GWAS) [14]. This approach enhances the mapping resolution, allowing for a more detailed understanding of the genetic factors at play. In the past few years, the application of GWAS in plant genetic studies, including in blueberry [15], has significantly contributed to the refinement of candidate gene regions, offering a more precise identification of the genetic elements influencing the traits under investigation.

As evidence builds around candidate genes through genetic studies, it becomes rational to perform functional characterization of these acyltransferase genes. Current methods for functional characterization of candidate genes in blueberries rely on the use of transient expression or stable transformation [16, 17]. Developing a stable transformation protocol in blueberry is very challenging because blueberry is recalcitrant to transformation [18]. Also, this method requires several years for the plant to develop and produce fruits that can be evaluated. In contrast, transient transformation is a rapid method that can be applied directly to the fruit [16, 19]. To date, virus-induced gene silencing (VIGS) has been used in blueberry to transiently silence the anthocyanidin synthase (ANS) a gene involved in anthocyanin biosynthesis [16]. The method was applied in developed fruits to visually assess the repression of anthocyanin accumulation (evidenced by green tissues). However, assessing the function of genes involved in the accumulation of metabolites or other fruit characteristics (e.g. texture) that do not results in the appearance of visual phenotype, like acylated anthocyanins, requires silencing of the target gene in the whole fruit, such that enough material can be processed for gene expression and biochemical analysis. Such method has not been established for blueberry.

To advance research toward candidate gene analysis for health-related bioactives in blueberry, this study used GWAS to identify QTL and candidate genes controlling ACN and CGA accumulation. In addition, a modified VIGS protocol that induce silencing in the whole fruit was established to perform functional validation of the candidate genes controlling acylation. This approach seeks to fill the gap in understanding the genetic intricacies governing the accumulation of these important bioactive compounds in blueberries and advance methods for rapid functional characterization of gene of interest.

## Results

### Metabolite profiling of blueberry accessions

A total of 20 individual ACN glycosides were identified by LC–MS, and quantified by HPLC analyses over a three-year period, except petunidin-3-arabinoside (Pet_arab), which was detected only in 2017 and 2018. The ACN profiles observed in this study align closely with those reported previously [3, 4, 6, 12]. Extensive phenotypic variation persisted for ACN and CGA, over three consecutive years (2017–2019) (Table S1). Across the individuals, the variability for individual ACN, CGA, and TotalACN content (the sum of 6 acylated and 13 non-acylated anthocyanins) was substantial, with over 5- and 23-fold variation for TotalACN, and CGA, respectively (Table S1).

**Figure 1:**
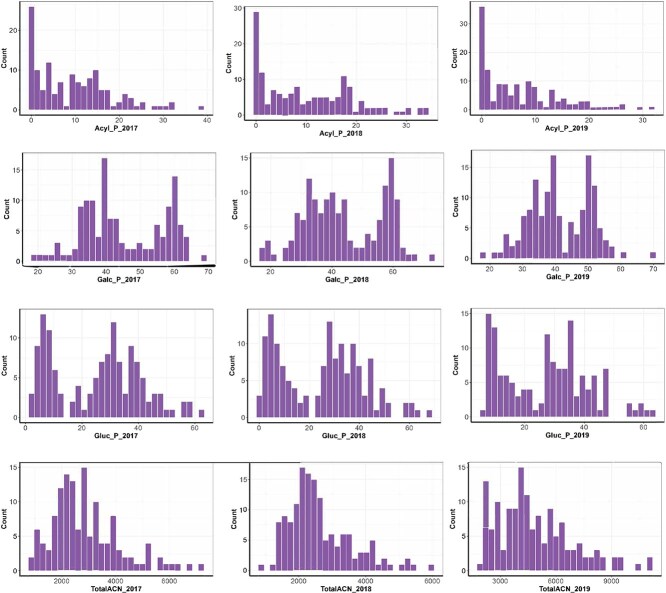
Anthocyanin content variation. Distribution of percentage acylated ACN (Acyl_P), percentage of galactoside ACN (Galc_P), percentage of glucoside ACN (Gluc_P) and totalACN (ug/g, fresh weight) over 3 years (2017–2019).

The histogram distribution of the metabolites evaluated here indicated a near-normal distribution for TotalACN and CGA, while individual ACN showed a bimodal or highly skewed pattern with skewness toward lower values (Fig. 1, Fig. S1). ACN composition, including acylated and non-acylated ACN, exhibited a bimodal distribution (Fig. 1). These skewed and bimodal distributions suggest that the ACN composition is influenced by a few genes, with minimal environmental effects.

**Table 1:** SNPs significantly associated with acylation of anthocyanin

**Trait**	**Marker ID**	**Model**	**Threshold**	**Chr**	**Pos**	**Score**	**Ref**	**Alt**	**R2**	**pval**
Acyl_P_2017	Chr02_11 929 791	1-dom-ref	6.14	2	11 929 791	7.74	A	G	2.05	0.10
Acyl_P_2017	Chr02_11 929 791	additive	6.14	2	11 929 791	8.20	A	G	2.64	0.06
Acyl_P_2017	Chr02_13 754 655	1-dom-ref	6.14	2	13 754 655	7.43	C	T	1.67	0.13
Acyl_P_2017	Chr02_13 754 655	additive	6.14	2	13 754 655	8.14	C	T	0.15	0.66
Acyl_P_2017	Chr02_16 087 027	1-dom-ref	6.14	2	16 087 027	6.29	A	G	0.40	0.47
Acyl_P_2017	Chr02_6 411 891	1-dom-ref	6.14	2	6 411 891	6.56	C	T	2.45	0.07
Acyl_P_2017	Chr02_8 444 353	additive	6.14	2	8 444 353	7.76	G	A	0.40	0.46
Acyl_P_2017	Chr02_9 156 575	1-dom-ref	6.14	2	9 156 575	8.31	G	C	2.23	0.08
Acyl_P_2018	Chr02_8 444 353	additive	6.14	2	8 444 353	6.66	G	A	15.34	0.00
Acyl_P_2019	Chr02_11 929 791	1-dom-ref	6.14	2	11 929 791	7.71	A	G	2.88	0.04
Acyl_P_2019	Chr02_11 929 791	additive	6.14	2	11 929 791	7.71	A	G	2.29	0.07
Acyl_P_2019	Chr02_13 754 655	1-dom-ref	6.14	2	13 754 655	7.05	C	T	1.46	0.15
Acyl_P_2019	Chr02_13 754 655	additive	6.14	2	13 754 655	7.15	C	T	0.05	0.79
Acyl_P_2019	Chr02_16 087 027	1-dom-ref	6.14	2	16 087 027	6.21	A	G	0.00	0.99
Acyl_P_2019	Chr02_8 444 353	additive	6.14	2	8 444 353	7.48	G	A	2.12	0.08
Acyl_P_2019	Chr02_9 261 987	1-dom-ref	6.14	2	9 261 987	8.06	A	G	2.40	0.06
acylation_blue	Chr02_10 260 746	1-dom-ref	6.14	2	10 260 746	7.79	G	A	0.09	0.71
acylation_blue	Chr02_10 260 746	additive	6.14	2	10 260 746	6.72	G	A	0.09	0.71
acylation_blue	Chr02_11 929 791	additive	6.14	2	11 929 791	8.03	A	G	3.44	0.02
acylation_blue	Chr02_12 389 803	1-dom-ref	6.14	2	12 389 803	7.84	C	G	0.81	0.26
acylation_blue	Chr02_13 754 655	1-dom-ref	6.14	2	13 754 655	7.21	C	T	0.00	0.97
acylation_blue	Chr02_13 754 655	additive	6.14	2	13 754 655	7.10	C	T	0.34	0.47
acylation_blue	Chr02_8 441 459	1-dom-ref	6.14	2	8 441 459	8.00	C	G	0.04	0.80
acylation_blue	Chr02_8 444 353	additive	6.14	2	8 444 353	7.98	G	A	2.25	0.06
Arab_P_2017	Chr02_20 927 628	1-dom-alt	4.32	2	20 927 628	5.73	T	A	5.08	0.01
Arab_P_2018	Chr02_20 927 628	1-dom-alt	4.32	2	20 927 628	4.45	T	A	9.11	0.00
Arab_P_BLUE	Chr02_20 927 628	1-dom-alt	4.32	2	20 927 628	4.44	T	A	2.94	0.03
CGA_2017	Chr02_57 416 698	1-dom-ref	6.14	2	57 416 698	6.38	G	A	6.33	0.00
CGA_2017	Chr02_57 416 698	additive	6.14	2	57 416 698	6.38	G	A	6.18	0.00
Cyn_galc_2018	Chr02_56 742 668	1-dom-alt	4.32	2	56 742 668	5.47	T	C	0.01	0.91
Dp_ac_gluc_2017	Chr02_11 119 874	1-dom-ref	6.14	2	11 119 874	6.87	A	T	0.00	1.00
Dp_ac_gluc_2017	Chr02_12 389 803	1-dom-ref	6.14	2	12 389 803	6.48	C	G	0.08	0.74
Dp_ac_gluc_2017	Chr02_13 754 655	1-dom-ref	6.14	2	13 754 655	6.87	C	T	0.00	1.00
Dp_ac_gluc_2017	Chr02_13 754 655	additive	6.14	2	13 754 655	6.53	C	T	2.67	0.06
Dp_ac_gluc_2017	Chr02_17 655 490	1-dom-ref	6.14	2	17 655 490	6.86	T	A	2.26	0.08
Dp_ac_gluc_2017	Chr02_17 655 490	additive	6.14	2	17 655 490	6.21	T	A	2.34	0.07
Dp_ac_gluc_2017	Chr02_9 156 575	1-dom-ref	6.14	2	9 156 575	8.62	G	C	5.05	0.01
Dp_ac_gluc_2018	Chr02_11 119 874	1-dom-ref	6.14	2	11 119 874	10.13	A	T	0.00	1.00
Dp_ac_gluc_2018	Chr02_11 929 791	additive	6.14	2	11 929 791	8.18	A	G	0.21	0.57
Dp_ac_gluc_2018	Chr02_12 389 803	1-dom-ref	6.14	2	12 389 803	9.36	C	G	0.01	0.88
Dp_ac_gluc_2018	Chr02_13 754 655	1-dom-ref	6.14	2	13 754 655	9.39	C	T	0.04	0.80
Dp_ac_gluc_2018	Chr02_13 754 655	additive	6.14	2	13 754 655	9.05	C	T	0.01	0.92
Dp_ac_gluc_2018	Chr02_15 965 405	1-dom-ref	6.14	2	15 965 405	7.77	T	A	3.88	0.02
Dp_ac_gluc_2018	Chr02_15 965 405	additive	6.14	2	15 965 405	6.48	T	A	2.84	0.04
Dp_ac_gluc_2018	Chr02_17 655 490	1-dom-ref	6.14	2	17 655 490	6.80	T	A	0.15	0.64
Dp_ac_gluc_2018	Chr02_6 751 501	additive	6.14	2	6 751 501	6.47	C	T	0.67	0.32
Dp_ac_gluc_2018	Chr02_7 051 818	1-dom-ref	6.14	2	7 051 818	7.85	C	T	0.44	0.42
Dp_ac_gluc_2018	Chr02_8 444 353	1-dom-ref	6.14	2	8 444 353	10.13	G	A	0.00	1.00
Dp_ac_gluc_2018	Chr02_9 261 987	additive	6.14	2	9 261 987	8.35	A	G	0.55	0.37
Dp_ac_gluc_2019	Chr02_10 245 912	1-dom-ref	6.14	2	10 245 912	10.66	C	G	2.08	0.08
Dp_ac_gluc_2019	Chr02_11 929 791	additive	6.14	2	11 929 791	8.40	A	G	0.42	0.44
Dp_ac_gluc_2019	Chr02_12 389 803	1-dom-ref	6.14	2	12 389 803	8.82	C	G	0.09	0.73
Dp_ac_gluc_2019	Chr02_13 754 655	1-dom-ref	6.14	2	13 754 655	9.87	C	T	0.05	0.79
Dp_ac_gluc_2019	Chr02_13 754 655	additive	6.14	2	13 754 655	9.41	C	T	0.00	0.93
Dp_ac_gluc_2019	Chr02_15 965 405	additive	6.14	2	15 965 405	7.35	T	A	3.55	0.02
Dp_ac_gluc_2019	Chr02_16 087 027	1-dom-ref	6.14	2	16 087 027	8.04	A	G	0.03	0.84
Dp_ac_gluc_2019	Chr02_17 655 490	1-dom-ref	6.14	2	17 655 490	7.31	T	A	0.70	0.32
Dp_ac_gluc_2019	Chr02_7 051 818	1-dom-ref	6.14	2	7 051 818	8.39	C	T	0.48	0.41
Dp_ac_gluc_2019	Chr02_9 261 987	additive	6.14	2	9 261 987	9.00	A	G	0.77	0.30
Dp_ac_gluc_BLUE	Chr02_11 119 874	1-dom-ref	6.14	2	11 119 874	10.13	A	T	0.00	1.00
Dp_ac_gluc_BLUE	Chr02_12 389 803	1-dom-ref	6.14	2	12 389 803	8.83	C	G	0.16	0.62
Dp_ac_gluc_BLUE	Chr02_13 754 655	1-dom-ref	6.14	2	13 754 655	9.47	C	T	0.68	0.30
Dp_ac_gluc_BLUE	Chr02_13 754 655	additive	6.14	2	13 754 655	8.81	C	T	0.10	0.69
Dp_ac_gluc_BLUE	Chr02_15 965 405	1-dom-ref	6.14	2	15 965 405	9.01	T	A	5.29	0.00

**Table 1:** Continued

**Trait**	**Marker ID**	**Model**	**Threshold**	**Chr**	**Pos**	**Score**	**Ref**	**Alt**	**R2**	**pval**
Dp_ac_gluc_BLUE	Chr02_15 965 405	additive	6.14	2	15 965 405	8.47	T	A	5.45	0.00
Dp_ac_gluc_BLUE	Chr02_17 655 490	1-dom-ref	6.14	2	17 655 490	8.21	T	A	1.08	0.19
Dp_ac_gluc_BLUE	Chr02_17 655 490	additive	6.14	2	17 655 490	7.04	T	A	0.86	0.25
Dp_ac_gluc_BLUE	Chr02_6 411 891	1-dom-ref	6.14	2	6 411 891	7.75	C	T	2.19	0.06
Dp_ac_gluc_BLUE	Chr02_8 444 353	1-dom-ref	6.14	2	8 444 353	10.13	G	A	0.00	1.00
Dp_ac_gluc_BLUE	Chr02_9 261 987	additive	6.14	2	9 261 987	8.05	A	G	0.27	0.52
Mv_ac_galc_2017	Chr02_10 245 912	1-dom-ref	6.14	2	10 245 912	9.93	C	G	2.18	0.09
Mv_ac_galc_2017	Chr02_11 929 791	additive	6.14	2	11 929 791	7.40	A	G	0.75	0.32
Mv_ac_galc_2017	Chr02_12 389 803	1-dom-ref	6.14	2	12 389 803	8.84	C	G	0.11	0.70
Mv_ac_galc_2017	Chr02_13 754 655	1-dom-ref	6.14	2	13 754 655	9.45	C	T	2.47	0.07
Mv_ac_galc_2017	Chr02_13 754 655	additive	6.14	2	13 754 655	8.71	C	T	0.37	0.48
Mv_ac_galc_2017	Chr02_15 965 405	1-dom-ref	6.14	2	15 965 405	11.46	T	A	13.44	0.00
Mv_ac_galc_2017	Chr02_15 965 405	additive	6.14	2	15 965 405	8.61	T	A	6.19	0.00
Mv_ac_galc_2017	Chr02_17 655 490	1-dom-ref	6.14	2	17 655 490	7.60	T	A	0.35	0.49
Mv_ac_galc_2017	Chr02_17 655 490	additive	6.14	2	17 655 490	6.21	T	A	0.14	0.67
Mv_ac_galc_2017	Chr02_7 051 818	1-dom-ref	6.14	2	7 051 818	8.47	C	T	0.32	0.51
Mv_ac_galc_2017	Chr02_9 156 575	1-dom-ref	6.14	2	9 156 575	11.73	G	C	9.67	0.00
Mv_ac_galc_2017	Chr02_9 261 987	additive	6.14	2	9 261 987	8.03	A	G	0.15	0.66
Mv_ac_galc_2018	Chr02_11 119 874	1-dom-ref	6.14	2	11 119 874	9.64	A	T	0.00	1.00
Mv_ac_galc_2018	Chr02_11 119 874	additive	6.14	2	11 119 874	8.85	A	T	0.04	0.80
Mv_ac_galc_2018	Chr02_12 389 803	1-dom-ref	6.14	2	12 389 803	9.82	C	G	1.00	0.22
Mv_ac_galc_2018	Chr02_12 389 803	additive	6.14	2	12 389 803	8.97	C	G	0.98	0.23
Mv_ac_galc_2018	Chr02_13 754 655	1-dom-ref	6.14	2	13 754 655	10.03	C	T	0.94	0.24
Mv_ac_galc_2018	Chr02_13 754 655	additive	6.14	2	13 754 655	9.79	C	T	0.30	0.51
Mv_ac_galc_2018	Chr02_15 965 405	1-dom-ref	6.14	2	15 965 405	7.69	T	A	2.30	0.06
Mv_ac_galc_2018	Chr02_15 965 405	additive	6.14	2	15 965 405	6.33	T	A	1.55	0.13
Mv_ac_galc_2018	Chr02_17 655 490	1-dom-ref	6.14	2	17 655 490	6.53	T	A	0.00	0.96
Mv_ac_galc_2018	Chr02_4 591 470	1-dom-ref	6.14	2	4 591 470	6.48	AC	AA	5.71	0.00
Mv_ac_galc_2018	Chr02_6 751 501	additive	6.14	2	6 751 501	7.47	C	T	0.54	0.37
Mv_ac_galc_2018	Chr02_6 752 522	1-dom-ref	6.14	2	6 752 522	7.68	A	C	0.31	0.50
Mv_ac_galc_2018	Chr02_8 444 353	1-dom-ref	6.14	2	8 444 353	9.64	G	A	0.00	1.00
Mv_ac_galc_2018	Chr02_9 261 987	additive	6.14	2	9 261 987	8.30	A	G	0.01	0.91
Mv_ac_galc_2019	Chr02_10 245 912	1-dom-ref	6.14	2	10 245 912	15.36	C	G	3.38	0.03
Mv_ac_galc_2019	Chr02_11 929 791	additive	6.14	2	11 929 791	11.44	A	G	0.88	0.26
Mv_ac_galc_2019	Chr02_12 217 810	1-dom-ref	6.14	2	12 217 810	13.32	A	T	4.04	0.02
Mv_ac_galc_2019	Chr02_13 754 655	1-dom-ref	6.14	2	13 754 655	13.41	C	T	0.22	0.57
Mv_ac_galc_2019	Chr02_13 754 655	additive	6.14	2	13 754 655	11.68	C	T	0.02	0.86
Mv_ac_galc_2019	Chr02_15 965 405	additive	6.14	2	15 965 405	6.83	T	A	0.53	0.39
Mv_ac_galc_2019	Chr02_16 087 027	1-dom-ref	6.14	2	16 087 027	10.18	A	G	0.08	0.74
Mv_ac_galc_2019	Chr02_17 655 490	1-dom-ref	6.14	2	17 655 490	8.53	T	A	0.13	0.66
Mv_ac_galc_2019	Chr02_17 655 490	additive	6.14	2	17 655 490	7.13	T	A	0.30	0.52
Mv_ac_galc_2019	Chr02_3 354 705	additive	6.14	2	3 354 705	6.69	A	G	0.01	0.92
Mv_ac_galc_2019	Chr02_3 809 515	1-dom-ref	6.14	2	3 809 515	7.90	G	C	0.51	0.39
Mv_ac_galc_2019	Chr02_7 051 818	1-dom-ref	6.14	2	7 051 818	11.77	C	T	0.11	0.69
Mv_ac_galc_2019	Chr02_7 051 818	additive	6.14	2	7 051 818	8.25	C	T	0.72	0.31
Mv_ac_galc_2019	Chr02_8 444 353	1-dom-ref	6.14	2	8 444 353	12.36	G	A	0.04	0.82
Mv_ac_galc_2019	Chr02_911 127	1-dom-ref	6.14	2	911 127	6.26	G	C	3.44	0.03
Mv_ac_galc_2019	Chr02_9 262 046	additive	6.14	2	9 262 046	10.19	T	G	0.42	0.44
Mv_ac_galc_BLUE	Chr02_10 245 912	1-dom-ref	6.14	2	10 245 912	12.63	C	G	1.00	0.21
Mv_ac_galc_BLUE	Chr02_11 119 874	additive	6.14	2	11 119 874	9.15	A	T	0.03	0.84
Mv_ac_galc_BLUE	Chr02_12 389 803	1-dom-ref	6.14	2	12 389 803	11.16	C	G	0.50	0.38
Mv_ac_galc_BLUE	Chr02_12 389 803	additive	6.14	2	12 389 803	9.09	C	G	0.30	0.50
Mv_ac_galc_BLUE	Chr02_13 754 655	1-dom-ref	6.14	2	13 754 655	12.44	C	T	0.31	0.49
Mv_ac_galc_BLUE	Chr02_13 754 655	additive	6.14	2	13 754 655	10.95	C	T	0.01	0.90
Mv_ac_galc_BLUE	Chr02_15 965 405	1-dom-ref	6.14	2	15 965 405	11.64	T	A	4.45	0.01
Mv_ac_galc_BLUE	Chr02_15 965 405	additive	6.14	2	15 965 405	9.31	T	A	4.72	0.01
Mv_ac_galc_BLUE	Chr02_17 655 490	1-dom-ref	6.14	2	17 655 490	8.77	T	A	0.51	0.37
Mv_ac_galc_BLUE	Chr02_17 655 490	additive	6.14	2	17 655 490	7.18	T	A	0.37	0.45
Mv_ac_galc_BLUE	Chr02_3 809 515	1-dom-ref	6.14	2	3 809 515	6.16	G	C	0.21	0.56
Mv_ac_galc_BLUE	Chr02_7 051 818	1-dom-ref	6.14	2	7 051 818	9.77	C	T	0.22	0.56
Mv_ac_galc_BLUE	Chr02_7 051 818	additive	6.14	2	7 051 818	7.42	C	T	0.77	0.27
Mv_ac_galc_BLUE	Chr02_9 156 575	1-dom-ref	6.14	2	9 156 575	11.68	G	C	0.70	0.30
Mv_ac_galc_BLUE	Chr02_9 261 987	additive	6.14	2	9 261 987	8.88	A	G	0.00	0.98
Mv_ac_gluc_2017	Chr02_11 119 874	1-dom-ref	6.14	2	11 119 874	7.79	A	T	0.00	1.00

**Table 1:** Continued

**Trait**	**Marker ID**	**Model**	**Threshold**	**Chr**	**Pos**	**Score**	**Ref**	**Alt**	**R2**	**pval**
Mv_ac_gluc_2017	Chr02_11 929 791	additive	6.14	2	11 929 791	6.38	A	G	0.49	0.42
Mv_ac_gluc_2017	Chr02_12 389 803	1-dom-ref	6.14	2	12 389 803	7.39	C	G	0.17	0.64
Mv_ac_gluc_2017	Chr02_13 754 655	1-dom-ref	6.14	2	13 754 655	7.79	C	T	0.00	1.00
Mv_ac_gluc_2017	Chr02_13 754 655	additive	6.14	2	13 754 655	7.79	C	T	0.11	0.70
Mv_ac_gluc_2017	Chr02_7 051 818	1-dom-ref	6.14	2	7 051 818	6.55	C	T	1.38	0.17
Mv_ac_gluc_2017	Chr02_8 444 353	additive	6.14	2	8 444 353	8.03	G	A	1.34	0.18
Mv_ac_gluc_2017	Chr02_9 156 575	1-dom-ref	6.14	2	9 156 575	9.23	G	C	4.12	0.02
Mv_ac_gluc_2018	Chr02_8 441 459	1-dom-ref	6.14	2	8 441 459	6.20	C	G	12.14	0.00
Mv_ac_gluc_2019	Chr02_10 245 912	1-dom-ref	6.14	2	10 245 912	8.72	C	G	1.73	0.12
Mv_ac_gluc_2019	Chr02_10 260 746	additive	6.14	2	10 260 746	7.61	G	A	0.37	0.47
Mv_ac_gluc_2019	Chr02_11 929 791	additive	6.14	2	11 929 791	7.53	A	G	1.06	0.22
Mv_ac_gluc_2019	Chr02_12 389 803	1-dom-ref	6.14	2	12 389 803	7.68	C	G	0.00	0.97
Mv_ac_gluc_2019	Chr02_13 754 655	1-dom-ref	6.14	2	13 754 655	8.24	C	T	0.92	0.25
Mv_ac_gluc_2019	Chr02_13 754 655	additive	6.14	2	13 754 655	7.96	C	T	0.00	0.99
Mv_ac_gluc_2019	Chr02_16 087 027	1-dom-ref	6.14	2	16 087 027	6.74	A	G	0.18	0.61
Mv_ac_gluc_2019	Chr02_6 411 918	1-dom-ref	6.14	2	6 411 918	7.97	T	A	3.91	0.02
Mv_ac_gluc_2019	Chr02_8 441 459	1-dom-ref	6.14	2	8 441 459	8.45	C	G	0.17	0.62
Mv_ac_gluc_2019	Chr02_8 444 353	additive	6.14	2	8 444 353	7.73	G	A	1.10	0.21
Mv_ac_gluc_BLUE	Chr02_10 260 746	additive	6.14	2	10 260 746	8.22	G	A	0.33	0.47
Mv_ac_gluc_BLUE	Chr02_11 119 874	1-dom-ref	6.14	2	11 119 874	8.84	A	T	0.10	0.69
Mv_ac_gluc_BLUE	Chr02_12 389 803	1-dom-ref	6.14	2	12 389 803	8.02	C	G	0.00	1.00
Mv_ac_gluc_BLUE	Chr02_13 754 655	1-dom-ref	6.14	2	13 754 655	8.48	C	T	0.07	0.74
Mv_ac_gluc_BLUE	Chr02_13 754 655	additive	6.14	2	13 754 655	8.43	C	T	0.04	0.80
Mv_ac_gluc_BLUE	Chr02_15 965 405	additive	6.14	2	15 965 405	7.03	T	A	2.97	0.03
Mv_ac_gluc_BLUE	Chr02_6 411 918	1-dom-ref	6.14	2	6 411 918	8.20	T	A	3.81	0.01
Mv_ac_gluc_BLUE	Chr02_6 752 522	additive	6.14	2	6 752 522	6.38	A	C	0.26	0.53
Mv_ac_gluc_BLUE	Chr02_8 441 459	1-dom-ref	6.14	2	8 441 459	9.06	C	G	0.10	0.70
Mv_ac_gluc_BLUE	Chr02_8 444 353	additive	6.14	2	8 444 353	8.92	G	A	1.26	0.16
Mv_gluc_2019	Chr02_58 141 575	1-dom-alt	4.32	2	58 141 575	5.05	C	T	13.35	0.00
Peo_galc_BLUE	Chr02_16 202 672	additive	6.14	2	16 202 672	6.56	T	G	5.68	0.00
Peo_gluc_2018	Chr02_2 572 121	1-dom-ref	6.14	2	2 572 121	8.25	G	A	10.18	0.00
Peo_gluc_2018	Chr02_2 572 121	additive	6.14	2	2 572 121	7.78	G	A	8.29	0.00
Peo_gluc_2018	Chr02_911 090	1-dom-ref	6.14	2	911 090	6.22	G	T	2.19	0.07
Peo_gluc_2018	Chr02_911 090	additive	6.14	2	911 090	6.38	G	T	4.46	0.01
TotalACN_2018	Chr02_46 752 005	additive	6.14	2	46 752 005	6.67	G	A	3.48	0.02
TotalACN_BLUE	Chr02_46 752 005	additive	6.14	2	46 752 005	6.38	G	A	14.65	0.00

*P-value for individual SNPs significance (*R*^2^) tested as a multiple QTL model using backward elimination.

**Figure 2:**
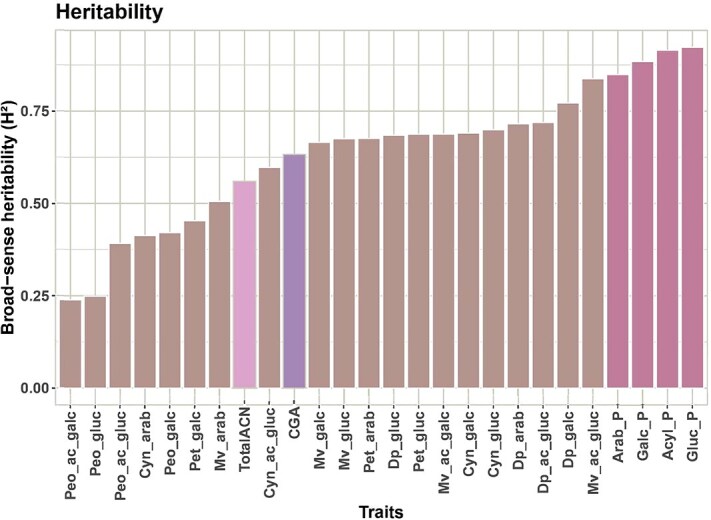
Estimates of broad sense heritability for raw concentrations of ACN and CGA, and percentage of arabinoside ACN (Arab-P), galactoside ACN (Galc_P), glucoside ACN (Gluc_P) and acylated ACN (Acyl_P) relative to the total ACN (TotalACN) content in 153 blueberry accessions over three years. Anthocyanidins abbreviation: Del, delphinidin; Mal, malvidin; Cyan, cyanidin; Pet, petunidin; Peo, peonidin. Sugar moieties and acylation abbreviations: arab, arabinoside; galc, galactoside; gluc, glucoside; ac or acyl, acylated.

Broad-sense heritability (H^2^) estimates indicated that individual ACN, TotalACN, and CGA have a spectrum of heritability ranging from moderate (≥40%) to high (>80%) levels, thereby highlighting that genetic factors play a significant role in the accumulation of these metabolites (Fig. 2). Notably, the ACN composition, and the percentages of acylated and glycosylated ACN had high heritability exceeding 80%, indicative of strong genetic factors influencing ACN composition. However, exceptions were noted for peonidin-6-acetyl-3-galactoside (Peo_ac_galc) and peonidin-6-acetyl-3-glucoside (Peo_gluc) that had relatively low heritability (<25%). These metabolites were detected at a relatively low amount, especially in 2017 and 2018, which could be due to environmental effects or lower resolution of the HPLC analysis at low metabolite concentration. This could explain the low heritability estimates for these ACNs.

Pearson correlation analysis revealed that individual ACN exhibit a grouping pattern based on their sugar moiety (glycosylation) or acyl-sugar-group (acylation) (Fig. S2). Notably, ACN with galactose and arabinose sugar moieties demonstrate a strong positive correlation, while those with glucose sugar moiety exhibit a robust positive correlation among themselves but show only slight or no correlation with arabinose or galactose-containing ACN (Fig. S2). Similarly, acylated ACN exhibits a positive correlation with glucoside-containing ACN. Interestingly TotalACN was highly correlated with most of the arabinoside and galactoside ACN forms, while the correlation with glucoside ACN was relatively lower. This indicates that glycosylation may have an effect on TotalACN. Regarding CGA, we observed a weak positive correlation with TotalACN and several individual ACNs (e.g. cyanidin-3-galactoside, Cyn_galc; peonidin-3-glucoside, Peo_gluc; peonidin-6-acetyl-3-glucoside, Peo_ac_gluc) (Fig. S2).

### Genome-wide association mapping

**Figure 3:**
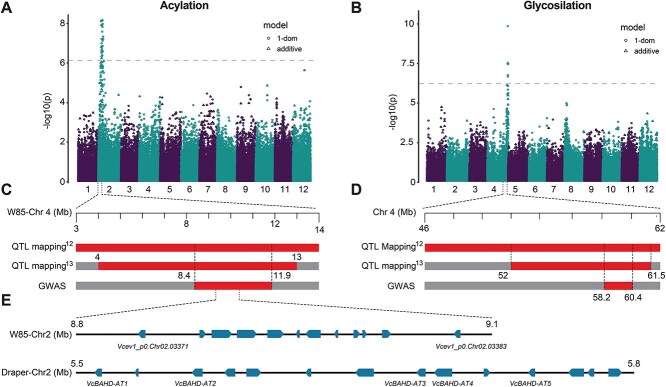
Mapping anthocyanin acylation and glycosylation loci. A) GWAS for anthocyanin acylation expressed as % of acylated anthocyanin (over total anthocyanin) using the Best Linear Unbiased Estimate (BLUE) model. B) GWAS for anthocyanin glycosylation expressed as % of glucosylated anthocyanin (over total anthocyanin) using the Best Linear Unbiased Estimate (BLUE) model. C) Schematic representation of overlapping loci associated with anthocyanin acylation identified on chromosome 2 in this study through GWAS and in previous studies through QTL mapping [12, 13]. D) Schematic representation of overlapping loci associated with anthocyanin glycosylation identified on chromosome 4 in this study through GWAS and in previous studies through QTL mapping [12, 13]. E) Schematic representation of the genomic region spanning BAHD candidate genes identified in the W85 genome and in the Draper genome. Arrowed boxes represent predicted genes and the direction of the arrow represent the direction of the coding sequence. The scheme was not drawn to scale.

GWAS was conducted using about 70 000 SNP markers. In total 326 significant SNPs associated with CGA, individual ACN concentrations, and ACN composition (expressed as % acylated and glycosylated ACN) were identified across all 12 chromosomes (Fig. 3A and B, Table 1, Table S2).

A significant stable QTL region was identified on chromosome 2, where 159 significant SNPs were detected (Table S2). These SNPs were associated with ACN acylation (%) and specific concentrations of acylated ACN, such as Mv_ac_gluc, Mv_ac_galc, and Dp_ac_gluc. This region, spanning from 1 to 18 Mb of the W85 genome [20], exhibited prominent peaks between 8.4 and 11 Mb. Further multiple QTL model analysis using backward elimination indicated that Chr02_8 444 353, in an additive model, explained the maximum phenotypic variance, accounting for 15% of the observed phenotypic variance among the significant SNPs (Table 1, Fig. S3). The analysis using the BLUE model [21] identified the region spanning position 8.4 to 11.9 Mb associated with ACN acylation (Fig. 3A and C, Table *S2*). This region overlaps with the QTL region associated with ACN acylation that has been identified by *[**12**,*  *13**]* in two biparental populations (Fig. 3C).

Another locus detected across the three years was mapped on chromosome 4 (Fig. 3B, Table 1, Table S2). This region harbored 52 significant SNPs associated with the glycosylation of anthocyanins (% galc, % gluc, and % arab) as well as individual anthocyanin concentrations, including Dp_gluc, Cyn_gluc, and Cyn_ac_gluc. The genomic region spanning these SNPs ranged from 52 to 61 Mb of the W85 genome chromosome 4, with peak SNPs identified around 60 Mb (Fig. 3B*,*  Table S2). A multiple QTL model analysis using backward elimination indicated that the SNP Chr04_60201524 explained the maximum percentage of the observed phenotypic variance, accounting for 27% among the significant SNPs (Table S2). To account for the genotype × year effect the GWAS was also carried out using the BLUE method. Using this approach, the region spanning position 58.19 to 60.4 Mb was associated with glycosylation of ACN (Gluc_P, Galc_P, Arab_P) (Fig. 3D). Interestingly, this genomic region overlaps with the previously identified region [12, 13] that was associated with anthocyanin glycosylation (Fig. 3D).

Investigation into SNP associations on other chromosomes revealed that these associations were not consistent across years, except for Dp_galc on chromosome 1, which was identified in two consecutive years (2017 and 2018). The SNPs linked to Dp_galc on chromosome 1 were located at 47 Mb of the W85 genome, with the individual SNP Chr01_47148938 explaining up to 20% of the phenotypic variance (Table *S2*). Additionally, the detection of Pet-gluc and MV-gluc at the same genome locus in a single year suggests that this location controls multiple metabolites.

Significant associations for CGA were identified on chromosomes 2, 3, 5, 6, 9, 10, and 11, while associations for TotalACN were observed on chromosomes 2, 3, 4, 5, 7, 8, and 12. However, none of these associations remained stable across years (Table *S2*), suggesting that TotalACN and CGA are quantitative traits. To explore potential year effects, a GWAS was conducted on the BLUE data, revealing novel associations, particularly for Peo_gluc_BLUE on chromosomes 3, 11, and 12, Peo_galc_BLUE on chromosome 6, Peo_arab_BLUE on chromosome 3, and CGA_BLUE on chromosome 9 (Table *S2*).

Overall, the GWAS study confirmed previous studies *[**12**,*  *13**]* that identified QTLs associated with ACN acylation in chromosome 2 and glycosylation in chromosome 4. Co-mapping of these QTLs across different mapping populations and a diversity panel in this study represents strong evidence for QTL validation.

### Identification of candidate genes controlling anthocyanin acylation

**Figure 4:**
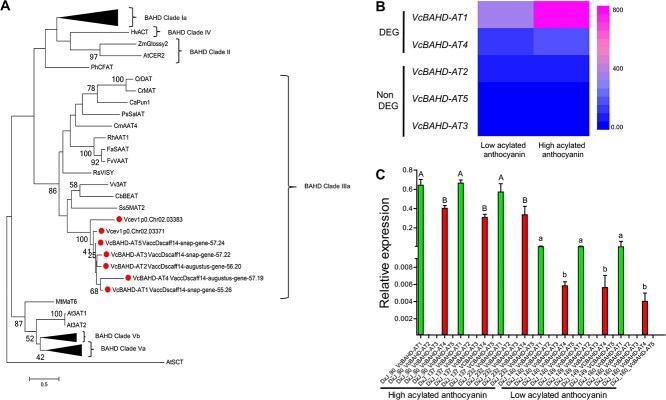
Characterization of BAHD acyltransferase genes in blueberry. A) Maximum Likelihood phylogenetic tree of BAHD acyltransferase from blueberry and other plant species. Bootstrap values represent percentages from 100 replicates. Dots indicate putative blueberry acyltransferase genes. The blueberry genes were extracted from the W85_v2 (Vcev1p0.Chr02.03371 and Vcev1p0.Chr02.03383) [12] and Draper v.1.0 (VcBAHD-AT1–5) **[**25] genome annotations. B) Heat map representing raw read counts of five BAHD acyltransferase genes in samples containing low and high acylated anthocyanin. C) Relative expression of *VcBAHD-AT1*, *VcBAHD-AT2*, *VcBAHD-AT3*, *VcBAHD-AT4*, and *VcBAHD-AT5* in D **×** J genotypes with high and low acylated anthocyanin. Data represent means ± SD (n = 3 technical replicates). Upper and lower-case letters, Aa, Bb, indicate statistically significant differences in the expression level of *VcBAHD-AT1* and *VcBAHD-AT4,* respectively. Statistical analysis was performed using two-tailed Student's *t*-tests (*p* > 0.05).

Previous work by [12] used the W85 genome to identify candidate genes involved in anthocyanin acylation in blueberry. The authors found two BAHD acyltransferase Vcev1_p0.Chr02.03371 and Vcev1_p0.Chr02.03383. Here for a more comprehensive gene mining analysis, the genomic region spanning 8.4 to 11.9 Mb in the W85 chromosome 2 and the corresponding region in the VaccDscaff14 of ‘Draper’ genome spanning 5.02 to 7.23 Mb were used to search for candidate genes for acylation. The analysis was carried out by integrating gene annotations, phylogenetics, and gene expression analysis. In the ‘Draper’ genome, five genes, VaccDscaff14-snap-gene-55.26 (*VcBAHD-AT1*), VaccDscaff14-augustus-gene-56.20 (*VcBAHD-AT2*), VaccDscaff14-snap-gene-57.22 (*VcBAHD-AT3*), VaccDscaff14-augustus-gene-57.19 (*VcBAHD-AT4*), and VaccDscaff14-snap-gene-57.24 (*VcBAHD-AT5*), were annotated as BAHD acyltransferase (Fig. 3E). In the W85 genome, the annotation of two putative BAHD acyltransferase genes, Vcev1_p0.Chr02.03371 and Vcev1_p0.Chr02.03383, was confirmed. The seven genes clustered with BAHD clade IIIa (Fig. 4A), along with *anthocyanin 3-O-glucoside-6″-o-acyltransferase* (Vv3AT), and *malony-CoA:anthocyanin 5-glucoside 4″’-O-malonyltransferase* (Ss5Mat2), which are known to function as anthocyanin acyltransferase in *Vitis vinifera* and *Salvia splendens,* respectively [22–24]. Closer inspection of the seven blueberry acyltransferases identified here highlighted that all except one (*VcBAHD-AT3*) harbor the HXXXD and DFGWG motifs that are needed for BAHD acyltransferases catalytic activity and binding of CoA [22]. Based on these results the ‘Draper’ genome harbors three extra clade IIIa BAHD. Next, transcriptome analysis was performed using available RNASeq data [12] representing samples with high-acylated (HA) anthocyanin and low-acylated (LA) anthocyanin (see section Material and methods). Among the five acyltransferase genes detected in the ‘Draper’ genome, only two genes; i.e*. VcBAHD-AT1* and *VcBAHD-AT4* were differentially expressed, with both genes being upregulated in HA samples (Fig. 4B, Table S3). The expression level of *VcBAHD-AT2* was not significantly different between HA and LA samples while *VcBAHD-AT4* and *VcBAHD-AT5* were not expressed. RT-qPCR validated the results of the RNASeq analysis (Fig. 4C). Thus, the two BAHD acyltransferase genes (*VcBAHD-AT1* and *VcBAHD-AT4*) represent the best candidate to control anthocyanins acylation in blueberry and were used for downstream functional analysis.

### Knockdown of *VcBAHD-AT1* and *VcBAHD-AT4* suppress the acylation of anthocyanins in blueberry

**Figure 5:**
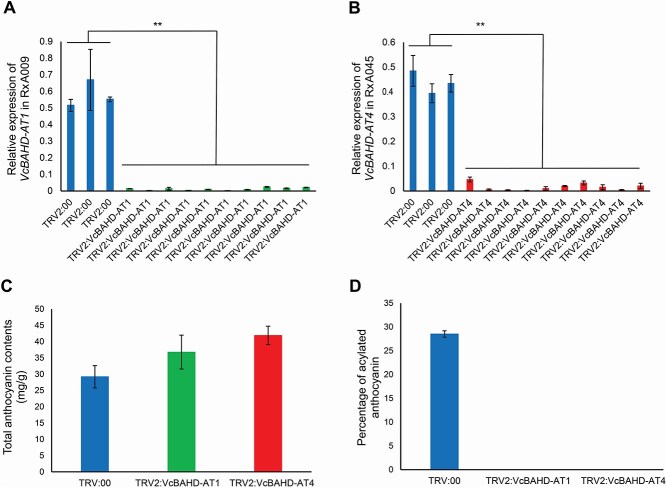
VIGS-induced suppression of *VcBAHD-AT1* and *VcBAHD-AT4* in blueberry breeding lines R × A009 and R × A045. A) Relative expression of *VcBAHD-AT1* in fruit infiltrated with TRV2:00 (empty) and TRV2:VcBAHD-AT1. B) Relative expression of *VcBAHD-AT4* in fruits infiltrated with TRV2:00 and TRV2:VcBAHD-AT4. Data in (A) and (B) represent means ± SD (n = 3 technical replicates). Statistical analysis was performed using two-tailed Student's *t*-tests (*p* > 0.05). C) Total anthocyanin contents in blueberry infiltrated with TRV:00 (empty), TRV2:VcBAHD-AT1 and TRV2:VcBAHD-AT4. D) Percentage of acylated anthocyanin contents in fruit infiltrated with TRV:00 (empty), TRV2:VcBAHD-AT1 and TRV2: VcBAHD-AT4. Data in (C) and (D) represent means ± SD (n = 3 technical replicates).

To test whether the *VcBAHD-AT1* and *VcBAHD-AT4* control the acylation of anthocyanin in blueberry, VIGS was used to suppress the expression of *VcBAHD-AT1* and *VcBAHD-AT4* in blueberry fruits. The experiment was carried out using two breeding lines R × A 009 and R × A 045 that have a higher % of acylated anthocyanin (Fig. S4). The tobacco rattle virus (TRV)-based VIGS construct specifically targeted the non-conserved region of *VcBAHD-AT1* and *VcBAHD-AT4* (Fig. S5). Real-time qPCR expression analysis revealed that transcripts of *VcBAHD-AT1* and *VcBAHD-AT4* were significantly reduced in blueberry fruits infiltrated with TRV2:*VcBAHD-AT1* and TRV2:*VcBAHD-AT4* compared to blueberry infiltrated with TRV2:00 (negative control) (Fig. 5A and B). This result indicated that *VcBAHD-AT1* and *VcBAHD-AT4* were silenced in R × A 009 and R × A 045 plants. To investigate potential off-target silencing, RT-qPCR was performed for all five acyltransferase genes in samples infiltrated with TRV2:*VcBAHD-AT4* (R × A 045) and samples infiltrated with TRV2:*VcBAHD-AT1* (R × A 009). The expression of the *VcBAHD-AT4* in samples infiltrated with TRV2:*VcBAHD-AT1* was not significantly different from the control, while the expression of *VcBAHD-AT1* was significantly reduced, as expected. The other three acyltransferases (*VcBAHD-AT2*, *VcBAHD-AT3,* and *VcBAHD-AT5*) were not detected in the control and treated samples (Fig. S6A). Similarly, the relative expression of *VcBAHD-AT1* in samples infiltrated with TRV2:*VcBAHD-AT4* and TRV2:00 did not change significantly, while the expression of *VcBAHD-AT4* in samples infiltrated with TRV2:*VcBAHD-AT4* was significantly reduced, as expected (Fig. S6B). Altogether, these results indicated that the silencing of one acyltransferase (e.g. *VcBAHD-AT1)* did not reduce the transcript level of another acyltransferase (e.g. *VcBAHD-AT4*) and vice versa, demonstrating that silencing was specific to each of these two genes. The results also confirmed that the other three acyltransferases are not expressed in these samples.

Next, to assess the effect of *VcBAHD-AT1* and *VcBAHD-AT4* silencing, HPLC was performed to determine the variation in total anthocyanin and acylated anthocyanin contents in samples infiltrated with TRV2:00, TRV2:*VcBAHD-AT1* and TRV2:*VcBAHD-AT4*. Total anthocyanin contents were not different between samples infiltrated with TRV2:00, TRV2:*VcBAHD-AT1* and TRV2:*VcBAHD-AT4* (Fig. 5C). However, acylated anthocyanins were not detected in samples infiltrated with TRV2:*VcBAHD-AT1* and TRV2:*VcBAHD-AT4*, while in the control samples infiltrated with TRV2:00, acylated anthocyanin reached 28.5% of the total anthocyanin contents, (Fig. 5D). These results demonstrated that *VcBAHD-AT1* and *VcBAHD-AT4* are required for the acylation of anthocyanins in blueberries.

## Discussion

The health benefits attributed to blueberry consumption drive interest from consumers, the scientific community and food industries. Studies indicated that anthocyanin and chlorogenic acid explain several health benefits attributed with blueberry consumption [1]. However, concentrations and chemical variants of ACN and CGA vary greatly in blueberries available to consumers, and the reported health benefits of blueberries may be highly dependent on levels of these bioactives. Thus, it is important to understand how levels of these compounds are controlled, and potentially use this knowledge to breed high ACN and CGA berries to maximize health benefit delivery to consumers. Given the importance of these bioactives, multiple studies assessed the genetic basis controlling their accumulation in blueberry fruits. Previous work highlighted that major QTLs control the increased content of chlorogenic acid and structural change of the anthocyanin-like acylation and type of glycosylation (with glucose, arabinose, galactose). To advance this work, in this study GWAS was used to validate previous QTLs and narrow the regions associated with these bioactives. In addition, RNASeq and VIGS were used to characterize genes controlling anthocyanin acylation. This is the first study in blueberry to perform a GWAS for these bioactives and to functionally characterize genes controlling anthocyanin acylation. The level of variation and heritability for total anthocyanins, chlorogenic acids, anthocyanin acylation, and glycosylation reported in this study were comparable to that reported in previous studies [6, 12, 25]. Consistent with previous QTL studies in blueberry, this study confirmed that two stable QTLs located on chromosome 2 and chromosome 4 control anthocyanin acylation and glycosylation. The higher resolution of the GWAS approach used in this study allowed narrowing of the regions associated with these QTLs. As expected, some QTLs detected in this study were not stable across years. Different factors including environmental factors [26] and the relatively small sample size (*N* = 153) may have contributed to the instability of these QTLs.

Anthocyanin glycosylation is an important enzymatic reaction that involves the addition of sugar moieties—glucose, arabinose, or galactose—to the anthocyanidin aglycones. This reaction is critical because it stabilizes the unstable anthocyanidin aglycone and it serves as a signal for the transport of the anthocyanin into the vacuoles [27–29]. The QTL in chromosome 4 associated with glycosylation explained up to 27% of the phenotypic variation. This QTL overlaps with a region previously associated with glycosylation and more specifically controlling the addition of glucose to the anthocyanidin aglycones [12, 13]. Overlapping the genomic regions associated with these QTLs across three studies highlighted that a 9.5 Mb region associated with anthocyanin glycosylation was reduced to a 2.2 Mb region (Fig. 3C). This region includes *Vcev1_p0.Chr04.11988* a UDP-glucosyl transferase previously identified as a potential candidate gene controlling this QTL. Efforts are ongoing to characterize this gene.

Acylation of anthocyanin is an enzymatic reaction that involves the addition of an acyl group to the glycosylated anthocyanin [30]. This reaction increases the stability of the anthocyanin color, reduces their sensitivity to thermal degradation, change of pH, and sulfite bleaching [31] and could increase the bioaccessibility and bioavailability of anthocyanins [6, 32–34]. This is particularly important because the bioavailability or level of absorption of the anthocyanin after consumption is relatively low. The QTL region associated with acylation mapped in chromosome 2 overlaps with acylation QTLs mapped in previous studies [12, 13]. Overlapping the genomic regions associated with these QTLs across three studies highlighted that a 9.0 Mb region, previously associated with anthocyanin acylation, was reduced to a 3.5 Mb region (Fig. 3C).

Identification of overlapping QTLs for anthocyanin acylation and glycosylation across multiple studies, germplasm and years provides strong evidence for validation of these QTLs. This represents the foundation for identifying candidate genes and to perform functional characterization. In this study functional characterization of the genes involved in acylation was performed. A previous study by Mengist *et al.* [12] identified two BAHD acyltransferases as the best candidate genes associated with this QTL. In this previous work, Mengist *et al.* used the W85 genome representing a diploid wild species (*V. caesariense*) as a reference to search for candidate genes. Although *V. caesariense* is closely related to tetraploid *V. corymbosum*, for a more comprehensive analysis of this region in this study, in addition to the W85 genome, the genome of ‘Draper’ (*V. corymbosum*), a commercially grown tetraploid blueberry cultivar was used. The ‘Draper’ genome more closely represents the genetic makeup of the tetraploid accessions (*V. corymbosum*) used for GWAS analysis. The results indicated that ‘Draper’ has five BAHD acyltransferases, three extra copies as compared to W85. The higher number of BAHD genes identified in ‘Draper’ could be the result of mis-predictions in the W85 genome or evolutionary divergence between the *V. caesariense* and the *V. corymbosum* genomes. BAHD genes are known to evolve relatively fast within each species [35].

Out of the five BAHD acyltransferases identified in the ‘Draper’ genome, two *VcBAHD-AT1* and *VcBAHD-AT4* were upregulated in the samples harboring the high acylation allele and were considered for functional characterization using VIGS. The other three genes were either not expressed or not differentially expressed. To exclude possible silencing of other orthologous BAHD genes, the W85 and Draper genomes were screened for additional BAHD genes. The best matches confirmed to be *VcBAHD-AT1–5*. Alternative matches in chromosome 12 had a very low identity (63.5%). The VIGS experiment demonstrated that silencing *VcBAHD-AT1* and *VcBAHD-AT4* resulted in a near-zero accumulation of acylated anthocyanin as compared to up to 28% of acylated anthocyanin accumulated in the control. These results suggest that *VcBAHD_AT1* and *VcBAHD_AT4* work together to carry out the acylation reaction. Although no firm support exists in the literature for such a mechanism, different analyses were carried out here to support this hypothesis. For instance, non-specific silencing could have led to false positive results, but real-time PCR demonstrated that the silencing was specific for *VcBAHD-AT1* and *VcBAHD-AT4.* Another possible explanation could be that due to mis-prediction, *VcBAHD-AT1* and *VcBAHD-AT4* coding sequences represent the same transcript/gene and the two silencing constructs were repressing the expression of the same gene. However, the two genes are not adjacent and, based on manual inspection of the raw transcriptome data, no reads spanning the two genes were identified. Also, both genes harbor the HXXXD and DFGWG motifs that are needed for BAHD acyltransferases catalytic activity and binding of CoA [22]. These observations support our hypothesis and future work is needed to verify the mode of action of these two genes in blueberry.

Multiple studies have clustered BAHD genes into seven different clades [36]. Most of the anthocyanin and flavonoid BAHDs belong to clade I [22, 36], but all seven BAHD acyltransferases identified here including *VcBAHD-AT1* and *VcBAHD-AT4* were placed in clade IIIa [22–24]. Clade III BAHD acyltransferases regulate acylation across a wide range of compounds including aliphatic alcohol, terpene, aliphatic amine, and alkaloid [37]. To date, only two clade III BAHD acyltransferases, *Vv3AT1* and *Ss5MAT*, have been proven to have anthocyanin acylation activity [22–24]. Clustering of *VcBAHD-AT1* to *VcBAHD-AT5* in clade III BAHD acyltransferases adds evidence to the involvement members of this clade in anthocyanin acylation and support the hypothesis indicating that multiple evolutionary routes have contributed to the emergence of anthocyanin acylation activity.

Developing methods for rapid functional characterization of candidate genes controlling traits related to fruit quality in blueberries is critical to advance molecular breeding for these traits. Methods used to test the function of candidate genes in blueberries include transient expression or stable transformation [16, 17]. While stable transformation represents the final proof to validate the function of a gene, developing stable transformation protocols in blueberry is challenging because it is recalcitrant to transformation [18]. In addition, transforming a plant to assess a gene function in the fruit can take 3–5 years. In contrast, transient expression methods like VIGS are relatively faster and can inform geneticists about which are the strongest candidate genes that can be tested using more time-consuming stable transformation methods. Previous work in blueberry used VIGS to silence *VcANS* a gene involved in anthocyanin biosynthesis [16]. In this experiment, the infiltration with the VIGS construct was performed when fruit were already formed and only skin tissue was infiltrated. Silencing of *VcANS* repressed the anthocyanin accumulation in a portion of the berry skin making it to appear green, while the portion of the skin where the gene was not silenced remained blue [16]. However to assess the function of a gene that does not result in a phenotype that can be visually been assessed like anthocyanin acylation or other fruit characteristics (e.g. texture) require silencing of the gene in the whole fruit, such that enough material can be collected to validate the effect of the silencing through RNA and metabolite analysis. In this study, we optimized the infiltration with TRV–VIGS vectors in the early stage of flower bud opening and fruit formation. This allowed us to successfully obtain silencing in the whole fruit. This method can be used in future studies to perform functional characterization of genes controlling other fruit characteristics in blueberries. Validation of the function of *VcBAHD-AT1* and *VcBAHD-AT4* through VIGS in this study establish a strong foundation to invest additional resources to test their using stable transformation methods.

Overall, this study identified and validated two major loci controlling anthocyanin glycosylation and acylation in blueberry and demonstrated the role of two clade III BAHD acyltransferases in anthocyanin acylation. These findings establish the foundation to develop marker-assisted selection strategy for this metabolite. Modulating anthocyanin acylation in new blueberry cultivars could potentially increase the absorption of this health-related bioactive. Also, the outcomes of this study expand our knowledge about the mode of action of BAHD acyltransferases in plants and provide tools to characterize other genes in this crop.

## Materials and methods

### Plant materials for GWAS analysis

The blueberry (*Vaccinium* spp.) accessions investigated in this study were sourced from the USDA National Clonal Germplasm Repository field collection in Corvallis, OR, USA. A comprehensive set of 153 tetraploid blueberry individuals, encompassing various cultivars and accessions, was used in the study (Table S4). Fully ripe (fully blue) berries were hand-harvested when over 50% of the berries in the bush were ripe. Fruits were collected for three seasons (2017, 2018, and 2019). After harvesting, the berries were stored at −80°C and shipped on dry ice to the Plants for Human Health Institute (PHHI), Kannapolis, North Carolina, United States. Berries were then stored at −80°C until processing.

### Chemical standards

Cyanidin-3-galactoside, cyanidin-3-glucoside, and malvidin-3-galactoside were sourced from Chromadex (Irvine, CA, USA), while delphinidin-3-glucoside was acquired from Cayman Chemicals (Ann Arbor, MI, USA). Delphinidin-3-galactoside, malvidin-3-glucoside, and petunidin-3-glucoside were obtained from Extrasynthese (Genay Cedex, France), and cyanidin-3-arabinoside along with peonidin-3-glucoside were procured from Polyphenols (Sandnes, Norway). Chlorogenic acid was purchased from Sigma-Aldrich (St. Louis, MO, USA). Organic solvents were HPLC grade. Distilled or double-distilled water was used to prepare the solutions.

### Extraction and quantification of anthocyanins and chlorogenic acid

The method for phenolic extraction followed a previously documented protocol [4, 12]. Frozen berries (10–30 g) from three replicates were homogenized (Waring blender 7012 G, Torrington, CT, USA). An aliquot of 3 g of the homogenized berries was transferred to a 30-mL centrifuge tube, mixed with 8 ml of 80% methanol in water (with 5% formic acid), and homogenized for 2 min. The homogenate was centrifuged at 4000 rpm for 2 min, and the supernatant was collected in a 25-ml volumetric flask. The residue was then subjected to two additional extractions: first with 8 ml of 80% methanol in water (5% formic acid) and second with 8 ml of 100% methanol. The combined supernatants were adjusted to a final volume of 25 ml. For HPLC analysis, 1 ml of each extract was diluted with an equal volume of a methanol–water–formic acid mixture (65:35:5) and filtered using a 0.22-μm PTFE membrane prior to HPLC- analysis.

For standard curve constructions, the actual concentrations of all standards were determined based on their purity. An eight-point calibration curve for each compound was established by injecting dilutions of the reference standard mix, resulting in linear standard curves with R^2^ values greater than 0.9997 ± 0.0007.

HPLC was performed on an Agilent 1260 HPLC equipped with a diode array detector (DAD) (Agilent Technologies, Santa Clara, CA, USA). The separation of ACN was executed using a Supelco C-18 column (25 cm × 4.6 mm × 5 μm) maintained at a column oven temperature of 30°C. The eluents consisted of water (with 5% formic acid, v/v) (A) and methanol (B), utilizing a precise gradient: 10%–20% B (0–5 min), 20%–25% B (5–20 min), 25%–30% B (20–25 min), 30%–35% B (25–30 min), followed by a steep increase to 35%–90% (30–43 min), and concluding with isocratic conditions at 90% B (43–46 min). The column was efficiently re-equilibrated for 4 minutes at 5% B, with a steady flow rate of 1 mL/min. Absorption readings were recorded at 520 nm for ACN and 280 nm for CGA. For those ACN lacking corresponding commercial standards, quantification was based on their respective aglycone–glucoside or -galactoside equivalents, ensuring accurate and reliable results.

### Phenotypic data analysis

To assess the extent of variation for ANT and CGA across accessions mean, minimum, maximum, and fold changes were computed and analyzed. The Best Linear Unbiased Estimate (BLUE) model was used to estimates the genotype mean, with both genotype and year treated as fixed factors. The broad-sense heritability was calculated through variance components using the Restricted Maximum Likelihood (REML) model, as outlined below:


$$ {H}^2=\frac{\partial_g^2}{\partial_g^2+\frac{\partial_{gy}^2}{y}+\frac{\partial_e^2}{ry}} $$

Here, ${\partial}_g^2$, ${\partial}_e^2$, and ${\partial}_{gy}^2$ correspond to the variance components of genotype, plot-to-plot variation of residuals, and [genotype × environment] interaction, respectively. The variable ‘y’ represents the number of environments (in this study, *y* = 3), and ‘*r*’ denotes the number of replications (*r* = 3).

The correlation between traits was computed using the Pearson Coefficient of Correlation, utilizing BLUE. Visualization of the correlation was facilitated through the R package corrplot [38].

### DNA extraction and SNP genotyping

DNA extraction, library preparation, and sequencing were performed at LGC (Gainesville, FL, USA) using a blueberry capture-seq platform that targets 50 000 probes/loci [39]. The pooled libraries of all individuals underwent demultiplexing and short reads were mapped to the blueberry reference genome (W85, P0 haplotype) [20] using BWA MEM alignment tool [40]. Variants were called using FreeBayes [41] for all accessions.

The resulting mapping and variant call file was filtered for minimum mapping quality (≥20), average coverage depth (≥30), maximum missing data across SNPs and individuals (≤30%), and number of alleles (max 2). The reference and alternative allele read depths for each SNP and individual were then extracted, with all steps performed using vcftools v.0.1.16 [42]. Genotype calling for tetraploid was performed using updog R package [43]. SNPs were coded as 0 for (AAAA), 1 for (AAAB), 2 for (AABB), 3 for (ABBB), and 4 for (BBBB). The ratio was derived for continuous genotyping data by dividing the read depth of the reference allele by the total read depth at each locus. A total of 69 503 high-quality SNP markers were utilized for the GWAS analysis.

### Genome-wide association analysis

The GWAS was performed using individual ACN content, TotalACN, ACN composition (expressed as % of total acylated or glycosylated ACN), and CGA content collected over three years (2017–2019). Each year’s data were analyzed separately using the R package GWASpoly [44]. Population structure and relationships were accounted by using a Q + K linear mixed model, where Q represents population structure and K denotes the matrix of kinship coefficients. SNP dosage was taken into consideration.

To identify SNPs significantly associated with target traits additive and complete dominance (‘1-dom’) models were tested. The Bonferroni multiple test was employed to establish the statistical significance LOD threshold for each trait, maintaining a 5% false-positive rate. GWASpoly [44] was used to generate Manhattan plots. The significance (*R*^2^) of SNPs was determined through a QTL model using backward elimination, while phenotypic variance was calculated via multiple regression analysis.

### RNA seq analysis

To identify candidate genes for anthocyanin acylation, transcriptome data released by [12] from genotypes belonging to a biparental population and representing the high-acylated (HA) and low-acylated (LA) anthocyanin alleles were used. The paired end raw reads from HA samples representing three biological replicates (DxJ_90: SRR19406445; DxJ_137: SRR19406443; DxJ_232: SRR19406448) and low-acylated (LA) samples representing three biological replicates (DxJ_140: SRR19406449; DxJ_149: SRR19406447; DxJ_160: SRR19406442) were downloaded from the NCBI database. To remove adapters, contaminations, and low-quality reads, the raw reads were filtered using the NGS QC toolkit [45]. The resulting high-quality paired-end clean reads were mapped to the blueberry genome Draper version 1.0 [46] using bowtie2 [47]. It should be noted that the RNASeq analysis using the W85 genome as a reference was previously conducted by our group [12] and was not performed in this study. SAMtools was used to sort and index the SAM files obtained from the bowtie2 for both HA and LA samples. The aligned sorted bam files were visualized in IGV [48]. RSEM was used to perform the quantification of reads and differential gene expression analysis was carried out using the edgeR [49, 50]. The gene-to-gene expression comparisons between HA and LA samples with *P*-value <0.05 were considered as significantly differentially expressed.

### RNA extraction and quantitative real-time PCR

Quantitative real-time PCR (qRT-PCR) was performed to validate the results of the RNASeq analysis and the VIGS experiments. To validate the RNAseq results for the ‘Draper’ samples, RNA for real-time PCR, was extracted from samples DSxJ_90, DSxJ_137, and DSxJ_232 representing the high-acylated group and samples DxJ_140, DxJ_149, and DxJ_160 representing the low-acylated group. Three biological replicates were used for each sample. Total RNA extraction was conducted using Spectrum Plant Total RNA Kit (Sigma-Aldrich, United States) and cDNA was synthesized using Verso cDNA Synthesis Kit (Thermo Fisher Scientific, USA) according to manufacturer’s instructions. qRT-PCR reactions were performed in 10-μl reaction volume following PowerUP SYBR Green Master Mix (Applied Biosystems, USA) instructions using a Roche LightCycler480 Real-time detection system (Roche Diagnostics, USA). Three technical replications were used for each biological replicate. Primer specificity was analyzed using melt curves analysis. Primer sequences used for this analysis are listed in Table S5. The statistical analysis was performed using two-tailed Student's *t*-tests (*P* > 0.05).

### Plant material for the VIGS experiment

Functional characterization of candidate genes associated with ACN acylation was performed using virus-induced gene silencing (VIGS). To perform the VIGS experiment, blueberry (*Vaccinium corymbosum)* breeding lines R × A 009 and R × A 045 were selected based on phenotypic data (Fig. S4). The two F_1_ clones had a % of acylated anthocyanin ranging from 15% to 22% comparable to the accessions evaluated in this study (15–39%) [12] carrying the QTL for high acylation. For the inoculation, plants were grown in pots in a growth chamber under the following conditions: 24°C/16°C (day and night), 70% RH, and 16 h photoperiod. The experiment was conducted at the Plants for Human Health Institute, Kannapolis. Floral buds and young green blueberry fruits were used for VIGS infiltration.

### Vector construction, VIGS infiltration, and validation

A TRV-based VIGS system was used to silence VaccDscaff14-snap-gene-55.26 (*VcBAHD-AT1)* and VaccDscaff14-augustus-gene-57.19 (*VcBAHD-AT4),* genes in two blueberry breeding lines R × A 009 and R × A 045. The coding sequences of *VcBAHD-AT1* and *VcBAHD-AT4* were obtained from GDV (vaccinium.org). Primer 3 (https://primer3.ut.ee/) was used to design primers (Table S5). PrimeStar GXL DNA polymerase (Takara bio USA) was used to amplify 353 and 316 bp fragments of non-conserved coding regions of *VcBAHD-AT1* and *VcBAHD-AT4,* respectively. To develop the TRV2:*VcBAHD-AT1* and TRV2:*VcBAHD-AT4* constructs, purified PCR products were ligated into the linearized pTRV2 vector using Infusion Snap Assembly Master mix (Takara bio USA) at EcoR1 and Kpn1 restriction sites. The TRV2:00 (empty) vector was used as a negative control. The TRV1, TRV2:00, TRV2:*VcBAHD-AT1*, and TRV2:*VcBAHD-AT4* were transformed into the *Agrobacterium tumefaciens* strain GV3101. Single colonies harboring each plasmid type were identified and confirmed by PCR and cultured overnight. The cells were first harvested by centrifugation and than resuspended in agroinfiltration buffer containing 150 μM acetosyringone, 10 mM MES, 10 mM MgCl_2_, pH 5.6 with a final concentration of OD_600_ to 2.0 and kept in the dark for 3 h at room temperature. The *Agrobacterium* cultures of TRV1 were mixed separately with TRV2:00, TRV2:*VcBAHD-AT1*, TRV2:*VcBAHD-AT4* at 1:1 (v/v) ratio and infiltrated into floral buds and also in green fruit until the fruit had a wet appearance. The injected plants were kept in the dark at 22°C for 3 days and then moved to a greenhouse.

To test the silencing of *VcBAHD-AT1* and *VcBAHD-AT4* in R × A 009 and R × A 045 plants infiltrated with TRV-VIGS vectors, qRT-PCR for each gene was performed as described above and compared with the TRV2:00 at four weeks after agroinfiltration. Fruits were collected at the fully ripening stage as indicated by the color (fully blue) as represented in Fig. S7. Each blueberry fruit was ground separately into a fine powder using mortar and pestle and divided into two aliquots/tubes, one used for qRT-PCR and the other one for HPLC assays. In total 100 berries for each line, including 50 representing the empty vector (TRV2:00) and 50 berries representing the vector with the gene (e.g. TRV2:*VcBAHD-AT1*) were tested by real-time. For each berries, three technical replicates were tested.

For the samples treated with TRV2:*VcBAHD-AT1* and TRV2:*VcBAHD-AT4,* fruit tissue from blueberries that were tested for having the gene silenced, were independently pooled into three replicates (3 g each), homogenized, and used for High-Performance Liquid Chromatography (HPLC) analysis. A similar approach was used for the samples treated with TRV2:00. HPLC was carried out as described above with three technical replicates for each of the pooled samples. Statistical analysis for real-time and HPLC was performed using two-tailed Student's *t*-tests (*P* > 0.05).

## Supplementary Material

Web_Material_uhaf041

## Data Availability

Phenotypic data used for QTL mapping were made available in Table S6. Genotypic data used for QTL analysis can be found on the Genome Database for Vaccinium at https://www.vaccinium.org/publication_datasets Accession # GDV24002.
