# Supplementary material for: Identification and functional characterization of BAHD acyltransferases associated with anthocyanin acylation in blueberry
Source: Hortic Res. 2025 Feb 10;12(5):uhaf041. doi: 10.1093/hr/uhaf041 (PMC11997424; doi:10.1093/hr/uhaf041)
Supplement: Web_Material_uhaf041 [file web_material_uhaf041.zip › Supplementary_Figures_S1-S7_v8_merged_edit.docx]

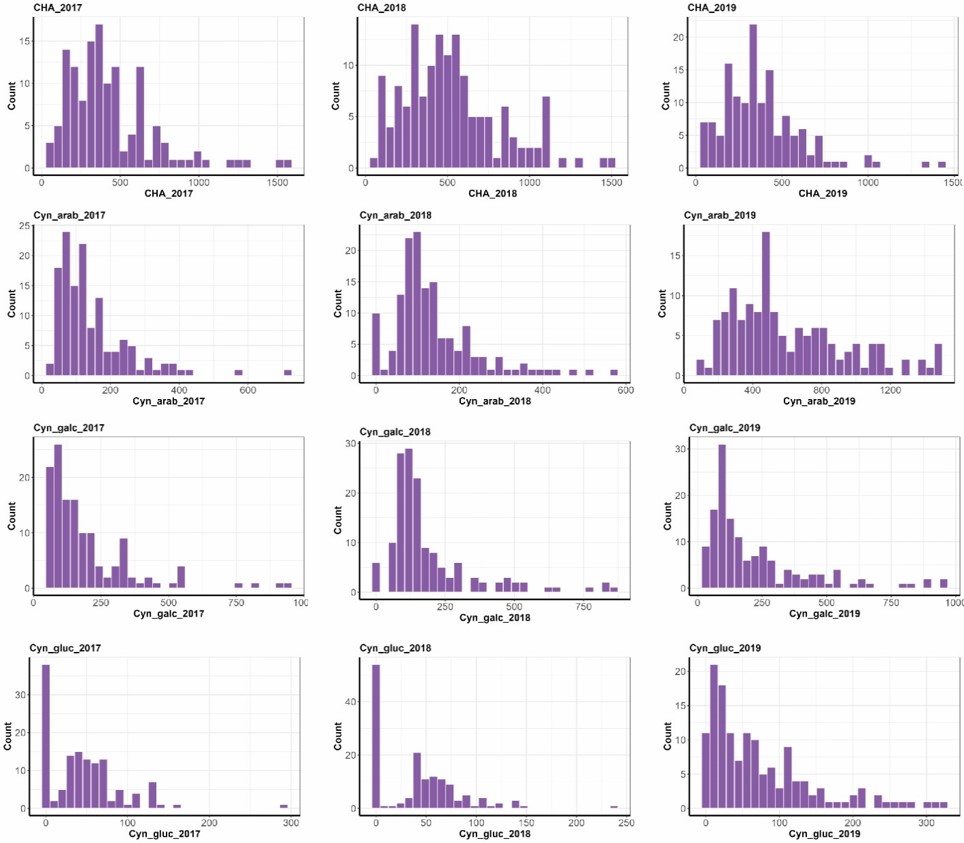


**Figure S1**. Anthocyanin and chlorogenic acid phenotypic variation. Phenotypic distribution of chlorogenic acid (CGA), cyanidin-3-arabinoside (Cyn_arab), cyanidin-3-galactoside (Cyn_galc), cyanidin-3-glucoside (Cyn_gluc) over 3 years (2017–2019). ACN content is expressed as µg/g, fresh weight.


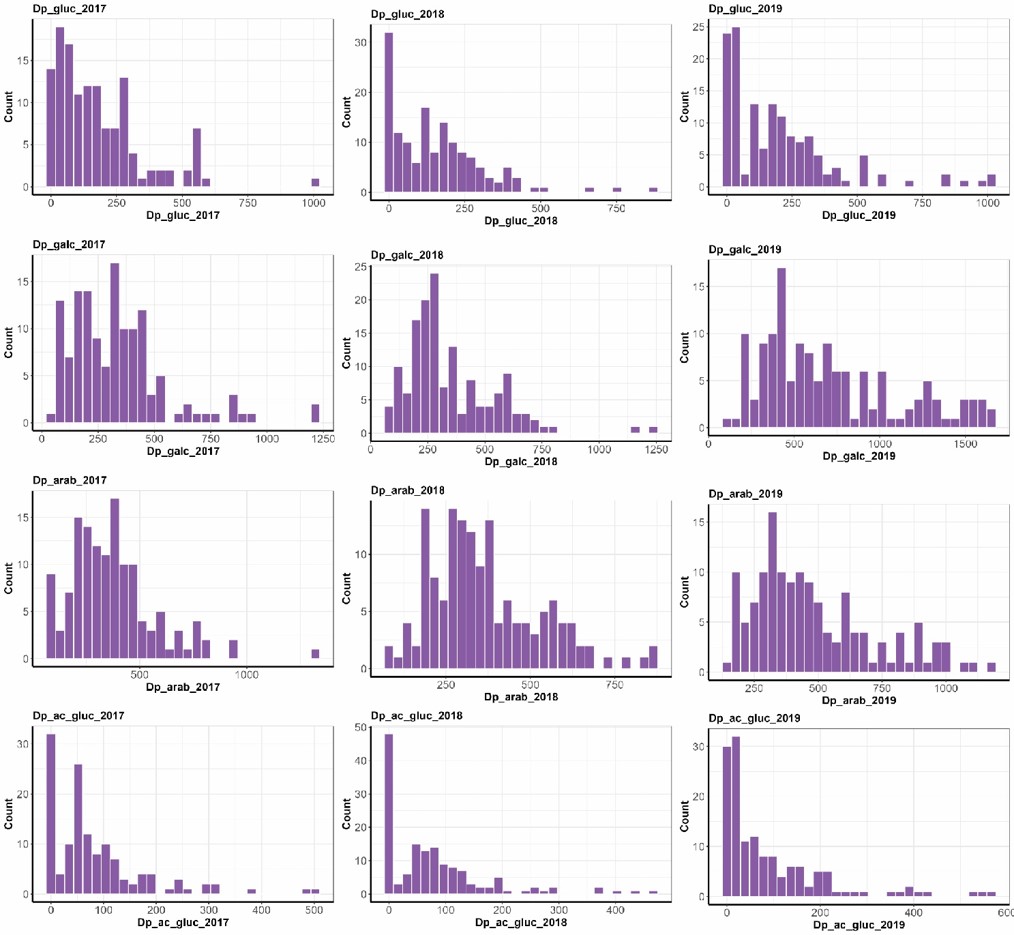


**Figure S1 continue**. Anthocyanin and chlorogenic acid phenotypic variation. Phenotypic distribution of delphinidin-3-glucoside (Dp_gluc), delphinidin-3-galactoside (Dp_galc), delphinidin-3-arabinoside (Dp_arab) and delphinidin-3-O-(6-acetoyl-3-glucoside) (Dp_ac_gluc) over 3 years (2017–2019). ACN content is expressed as ug/g, fresh weight.


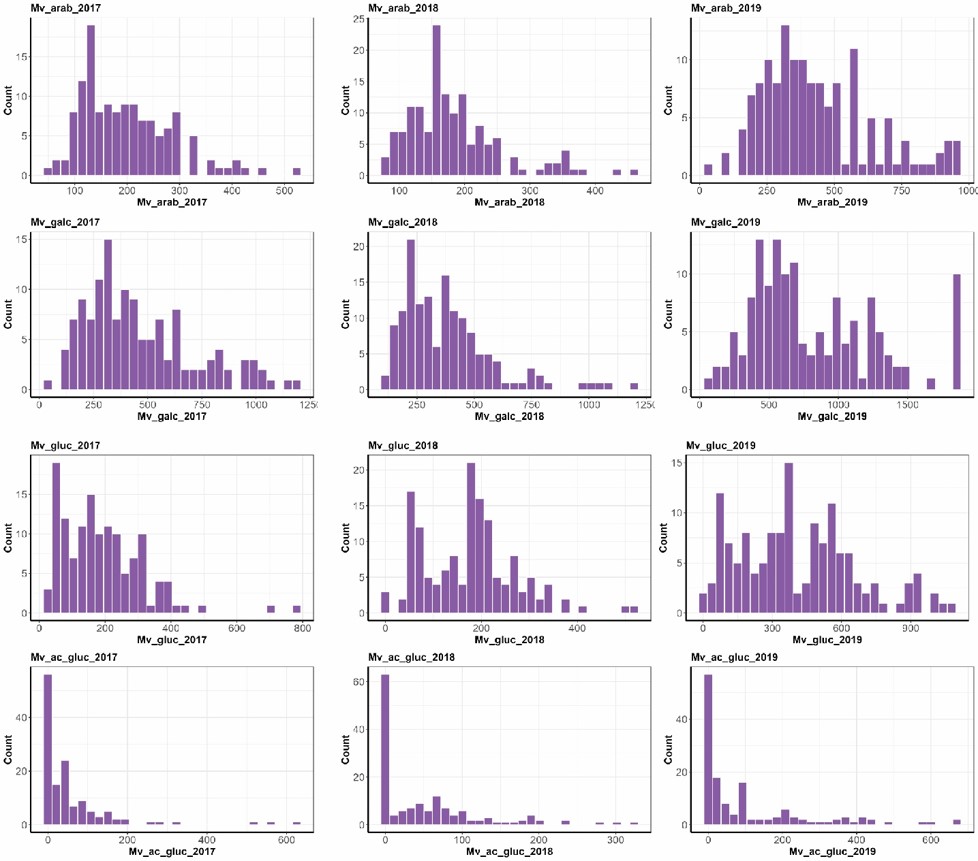


**Figure S1 continued**. Anthocyanin and chlorogenic acid phenotypic variation. Phenotypic distribution of malvidin-3-arabinoside (Mv_arab), malvidin-3-galactoside (Mv_galc), malvidin-3-glucoside (Mv_gluc) and malvidin-3-O-(6”-acetoylglucoside) (Mv_ac_gluc) over 3 years (2017–2019). ACN content is expressed as µg/g, fresh weight.


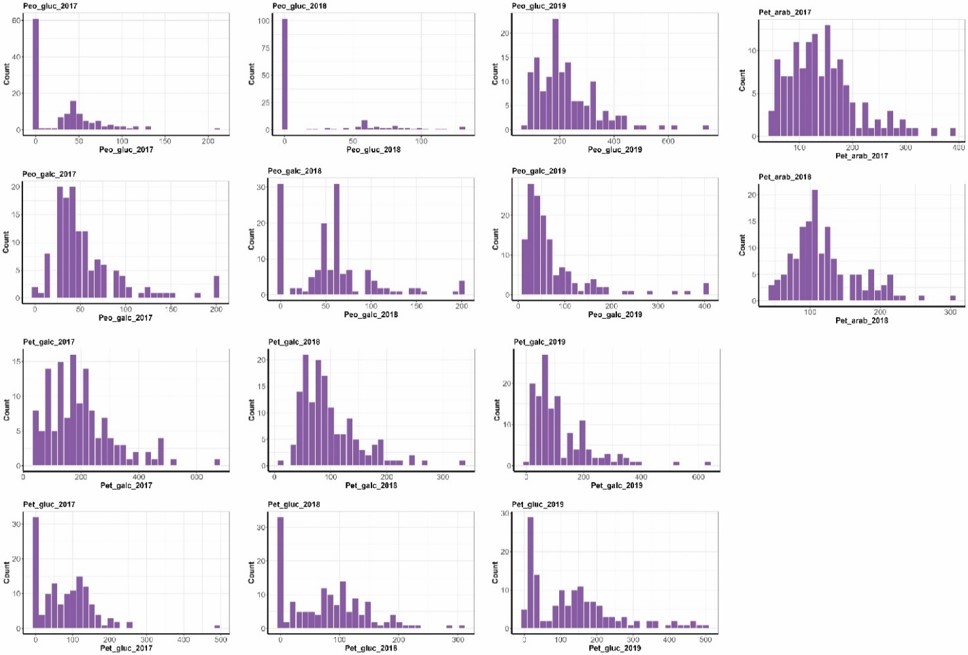


**Figure S1 continued**. Anthocyanin and chlorogenic acid phenotypic variation. Phenotypic distribution of peonidin-3-glucoside (Peo_gluc), peonidin-3-galactoside (Peo_galc), petunidin-3-arabinoside (Pet_arab), petunidin-3-galactoside (Pet_galc) and petunidin-3-glucoside (Pet_gluc) over 3 years (2017–2019). ACN content is expressed as µg/g, fresh weight.


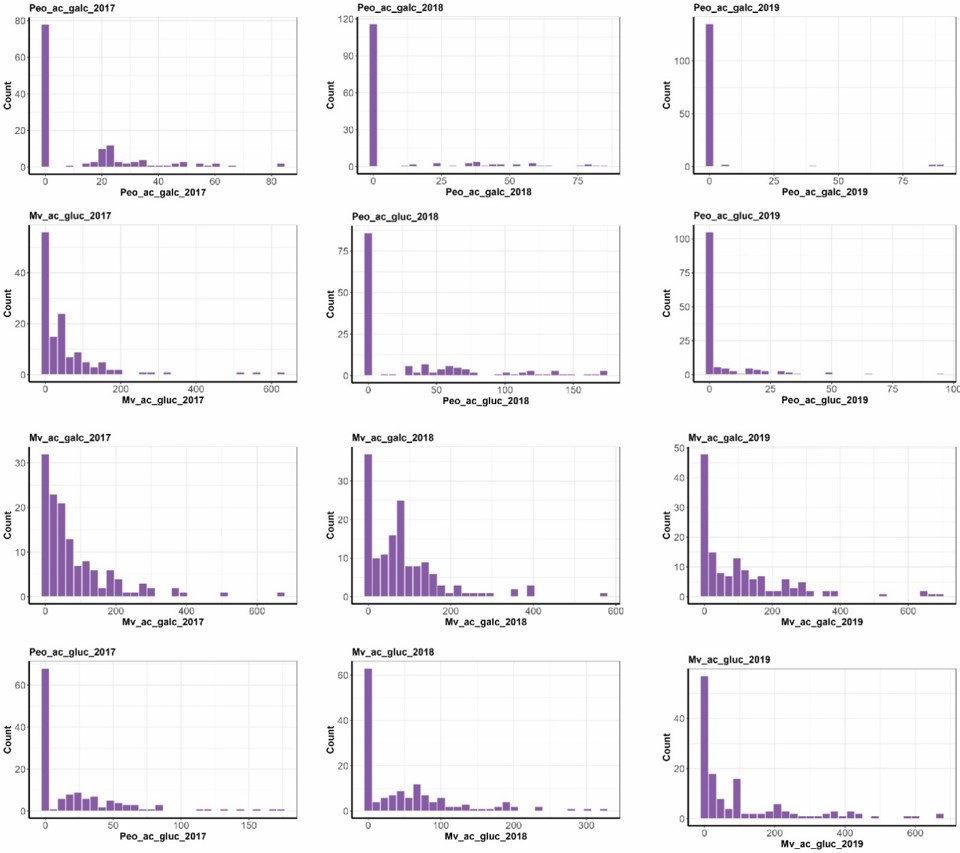


**Figure S1 continue**. Anthocyanin and chlorogenic acid phenotypic variation. Phenotypic distribution of peonidin-6-acetyl-3-galactoside (Peo_ac_galc), peonidin-6-acetyl-3-glucoside (Peo_ac_gluc), malvidin-6-acetyl-3-glucoside (Mv_ac_gluc), malvidin-3-O-(6”-acetoylgalactoside) (Mv_ac_galc) over 3 years (2017–2019). ACN content is expressed as µg/g, fresh weight.


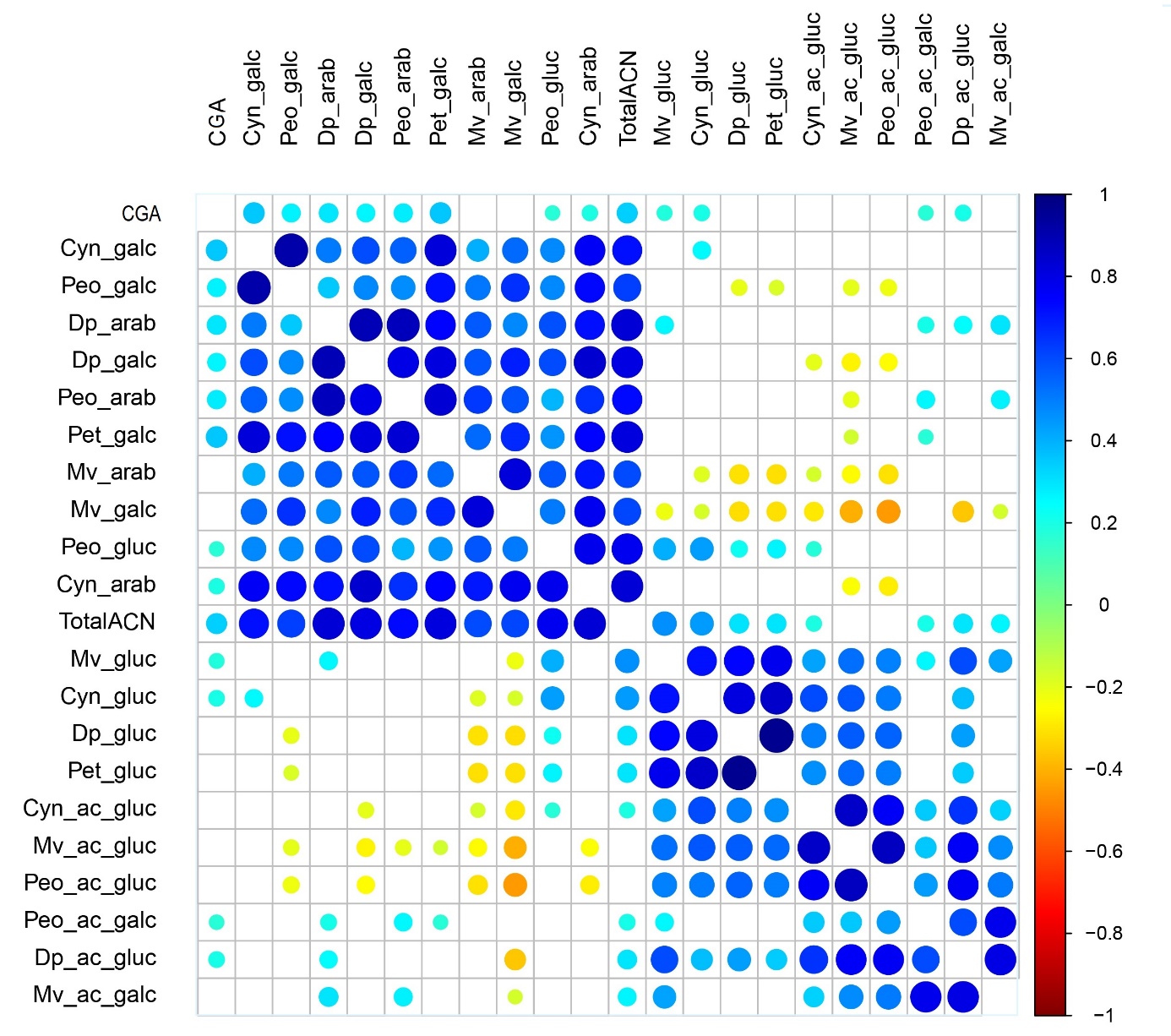


**Figure S2**. Pearson correlation between content of individual anthocyanin, chlorogenic acid and total anthocyanin.


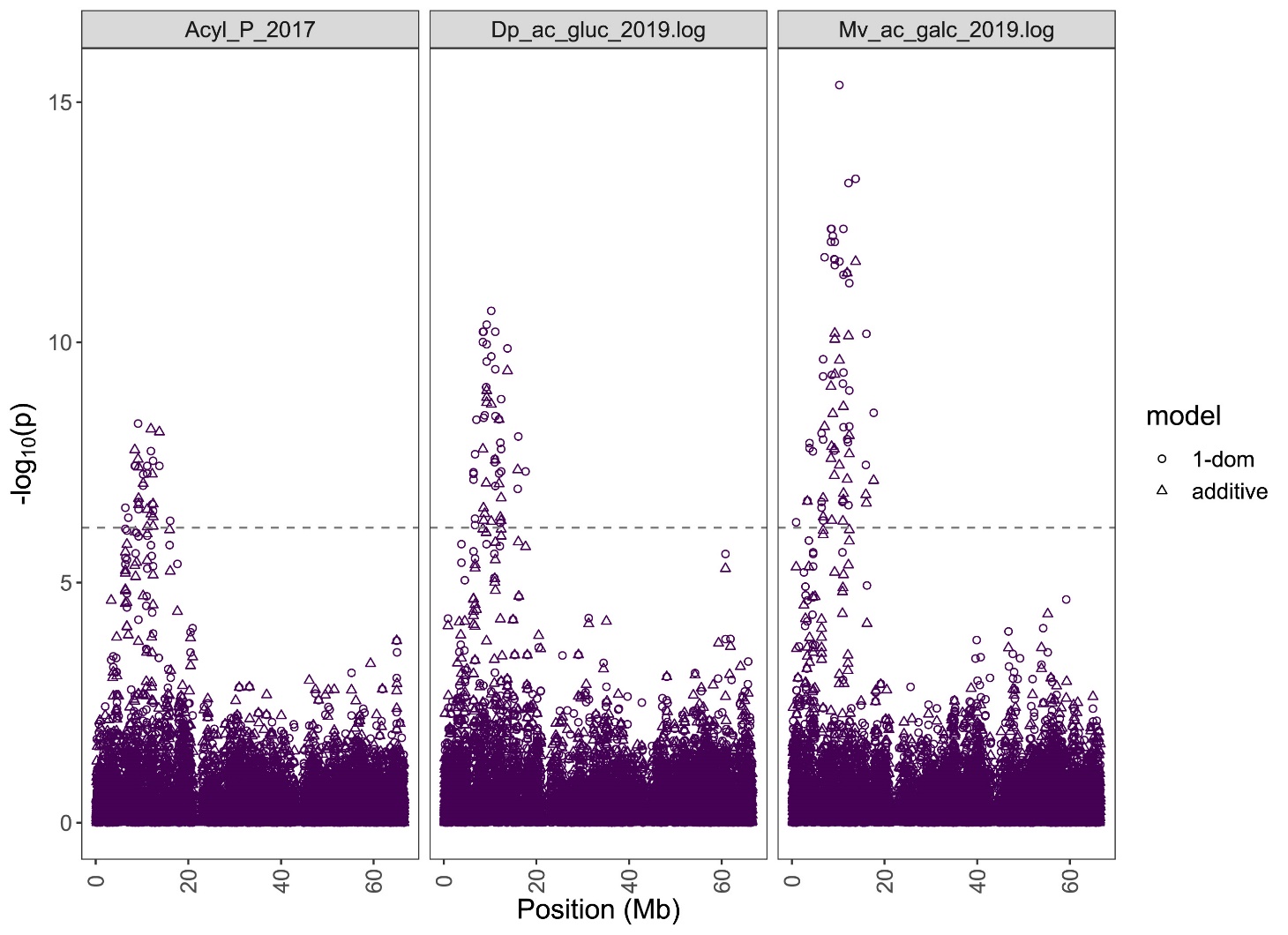


**Figure S3**. Manhattan plots showing significant SNPs associated with the percentage of acylated anthocyanins (Acyl_P_2017), and individual acylated anthocyanin concentrations (Dp_ac_gluc, and Mv_ac_galc) on chromosome 2.

**
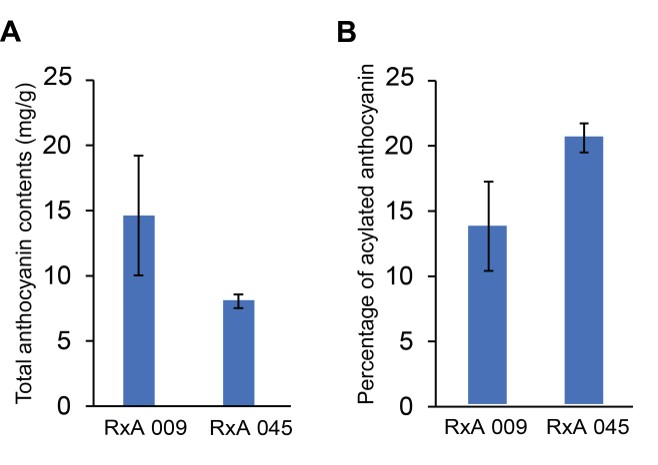
**

**Figure S4**. Total ACN content and percentage of acylated ACN in blueberry F_1_ genotype RxA 009 and RxA 045. These genotypes were used for the VIGS experiment.


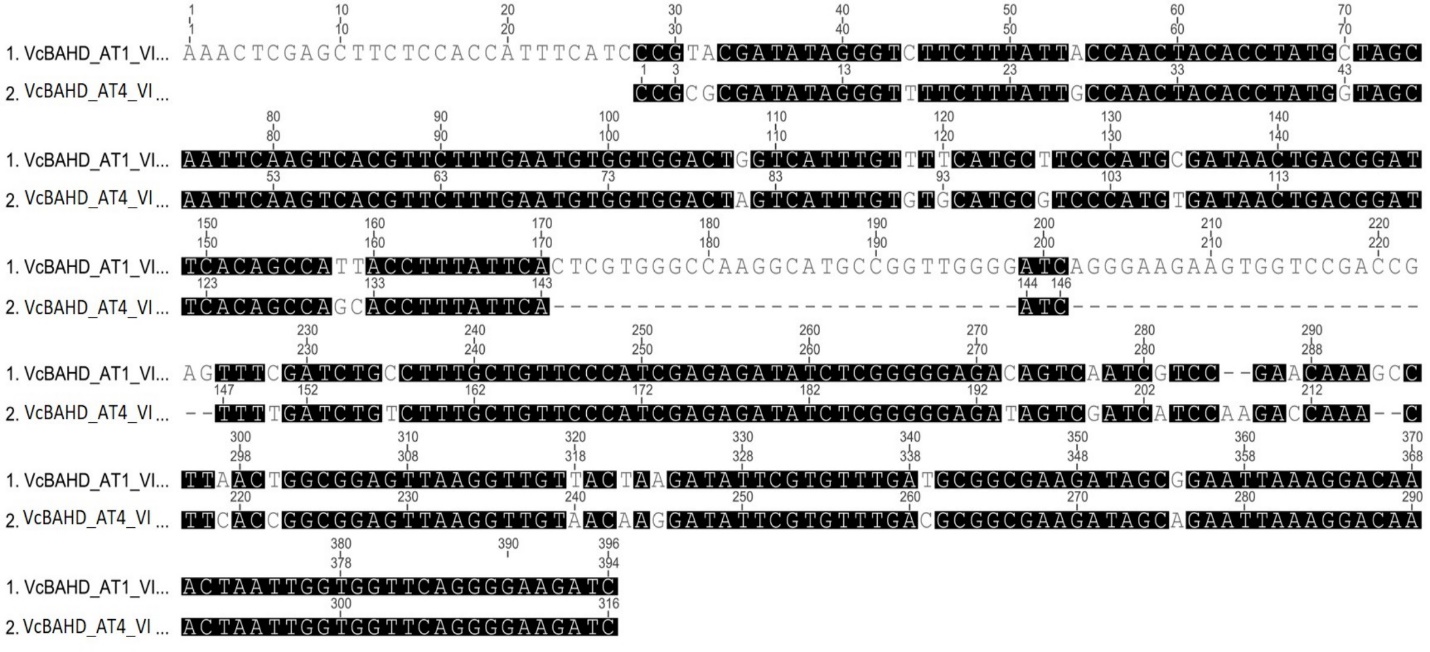


**Figure S5**. DNA sequence alignment of the *VcBAHD-AT1* and *VcBAHD-AT4* fragments used for VIGS*.*


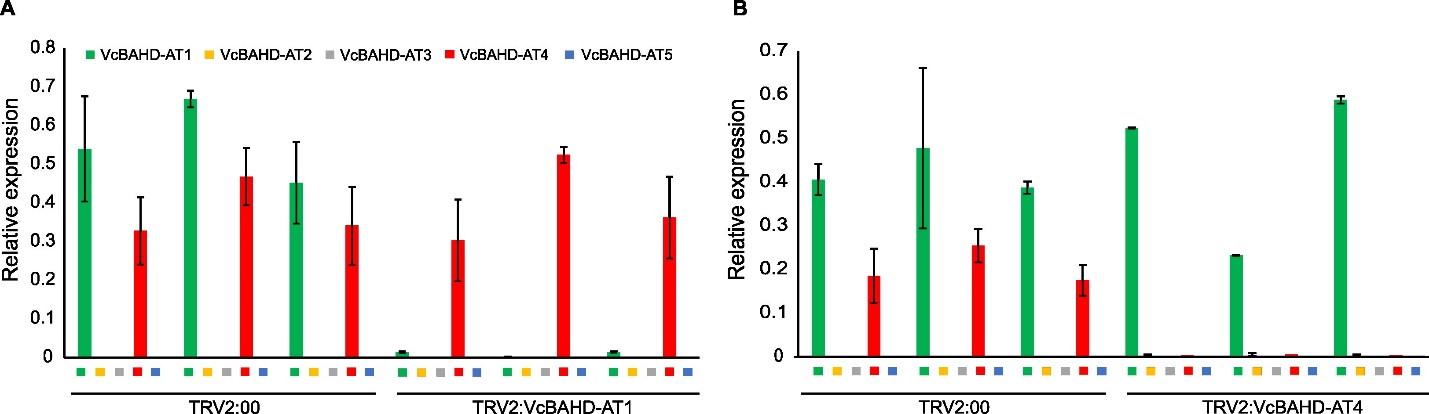


**Figure S6**. Relative expression of five acyltransferase genes in VIGS treated plants**.** A**)** Relative expression of *VcBAHD-AT1, VcBAHD-AT2, VcBAHD-AT3, VcBAHD-AT4* and *VcBAHD-AT5* in fruits infiltrated with TRV2:00 and TRV2:VcBAHD-AT1. B**)** Relative expression of *VcBAHD-AT1, VcBAHD-AT2, VcBAHD-AT3, VcBAHD-AT4* and *VcBAHD-AT5* in fruits infiltrated with TRV2:00 and TRV2:VcBAHD-AT4. Data in (A) and (B) represent means ± SD (n=3 technical replicates)


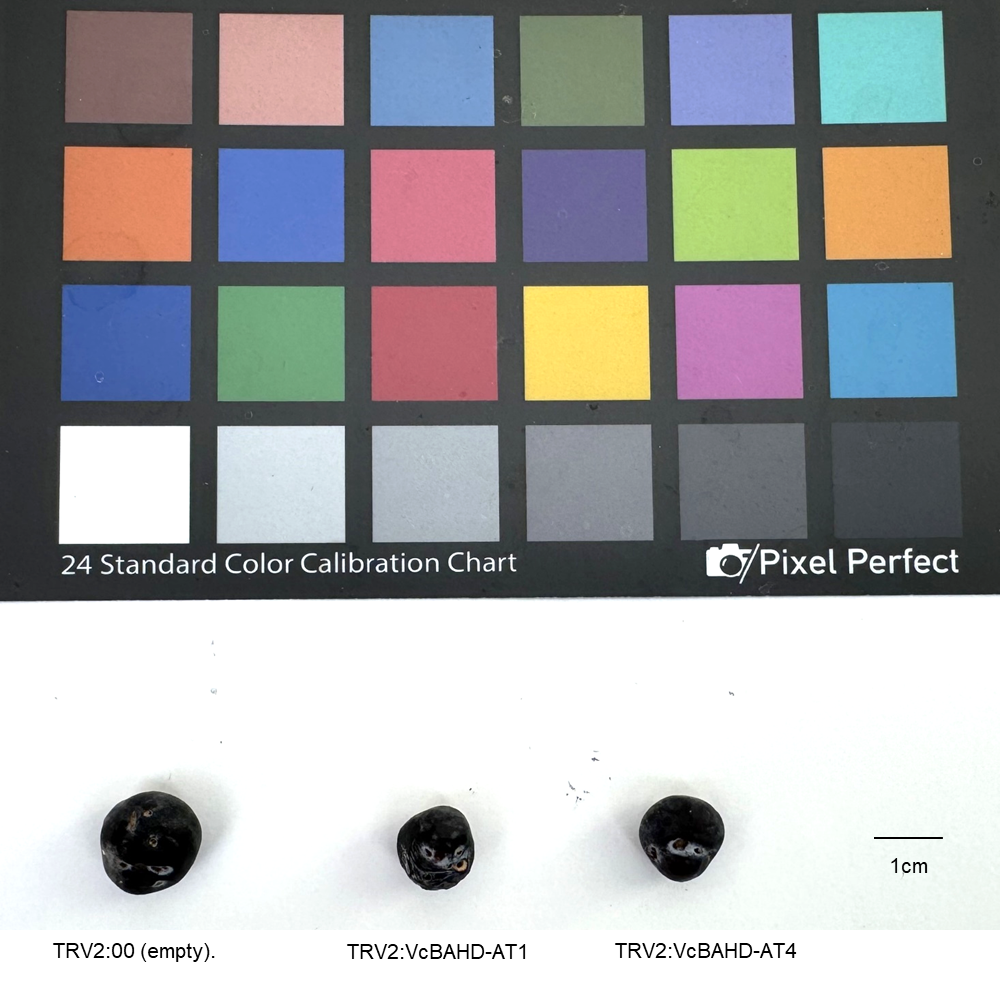


**Figure S7**. Phenotypic comparison of blueberries infiltrated with *TRV2:00* (empty vector), *TRV2:VcBAHD-AT1* and *TRV2:VcBAHD-AT4*.
